# A Computational Framework for Simulating Patient-Specific TMJ Biomechanics Using a Combined Multibody Dynamics and Finite Element Approach

**DOI:** 10.1007/s10439-026-04020-0

**Published:** 2026-03-02

**Authors:** Farhad Ahmadi, Shuchun Sun, Jichao Zhao, Jian Chen, Marshall B. Wilson, Brooke Damon, Yongren Wu, Konstantinia Almpani, Rachel Chung, Priyam Jani, Peng Chen, Elizabeth H. Slate, Janice S. Lee, Benedikt Sagl, Hai Yao

**Affiliations:** 1 Clemson-MUSC Joint Bioengineering Program, Department of Bioengineering, Clemson University, Clemson, SC USA; 2 Department of Oral Health Sciences, Medical University of South Carolina, Charleston, SC USA; 3 Department of Orthopaedics, Medical University of South Carolina, Charleston, SC USA; 4 Craniofacial Anomalies and Regeneration Section, National Institute of Dental and Craniofacial Research (NIDCR), NIH, Bethesda, MD USA; 5 Department of Statistics, Florida State University, Tallahassee, FL USA; 6 Competence Center Artificial Intelligence in Dentistry, University Clinic of Dentistry, Medical University of Vienna, Vienna, Austria; 7 Comprehensive Center AI in Medicine, Medical University of Vienna, Vienna, Austria

**Keywords:** Temporomandibular joint, Patient-specific modeling, Computer simulation, Orthognathic surgery

## Abstract

**Purpose:**

Biomechanical parameters of the temporomandibular joint (TMJ), such as joint contact forces and intra-articular stresses, are suggested to contribute to the development of temporomandibular joint disorders, but are impractical to measure. In this study, we present a computational framework for evaluating these parameters by integrating a function assessment system and a patient-specific modeling approach.

**Methods:**

The pipeline consists of acquiring patients' functional and morphological data and developing combined multibody dynamics and finite-element (MBD-FE) models for simulating their specific biting tasks. We demonstrate the approach in a pre-/post-orthognathic surgery scenario and present the measured and simulated outputs.

**Results:**

In a three-patient cohort of one Class I control and two surgical patients (one Class II and one Class III patient), surgery was accompanied by functional changes such as increased bite force capacity and shifts in muscle-usage during unilateral first premolar clenching that brought the surgical cases closer to the control case. Also, morphological measurements showed postoperative adaptations in condylar size and joint space. Simulations demonstrated that contralateral joint forces exceeded ipsilateral forces during unilateral biting and predicted regions of concentrated disc stress that coincided with regions of reduced joint gap and poorer articular congruency, highlighting how morphology-function interactions shape local mechanics.

**Conclusion:**

By unifying individualized functional inputs and subject-specific geometries, the framework provides a practical basis for patient-tailored assessment of biomechanical parameters and decision support in TMJ care.

**Supplementary Information:**

The online version contains supplementary material available at https://doi.org/10.1007/s10439-026-04020-0.

## Introduction

The temporomandibular joint (TMJ) is essential for functions such as mastication and speech. TMJ disorders (TMDs) affect about 12 million people in the U.S. [1], substantially impairing their quality of life. Since direct in vivo measurement of internal TMJ forces and stresses is impractical, due to the joint’s small size, complex structure, and deep location within the craniofacial region, computational approaches provide a practical route to estimate clinically relevant parameters.

Prior work has advanced TMJ modeling along complementary fronts. One line of work uses subject-specific musculoskeletal or numerical models to estimate muscle forces and joint loading during biting or to characterize mechanobehavioral exposure via metrics such as energy density and duty factors. These approaches illuminate how coordination and task demands relate to TMJ loading, but often simplify joint contact and soft-tissue mechanics, limiting inference about intra-articular stresses [2–6]. A second line emphasizes soft-tissue constitutive behavior within finite-element (FE) analyses, showing that modeling the disc with advanced material behaviors matters for local stress patterns by altering motion damping and stress-relaxation responses under prolonged clenching. This approach improves the fidelity of local mechanical responses but is typically applied to idealized geometries or simplified loading conditions, making it difficult to generalize to individual patients [7–9]. A third line examines surgical interventions to link morphology changes with loading, reporting shifts in stress or strain energy density under asymmetric occlusions and pre- and postoperative (pre-op and post-op) conditions; however, these studies commonly rely on generic or simplified boundary conditions and loading[10–12]. A fourth line of research employs integrated multibody dynamics (MBD) and FE modeling to capture concurrent joint dynamics and soft-tissue deformation, enabling the computationally efficient analysis of internal stresses. However, these studies rely on generic anatomies and idealized mastication tasks, leaving a gap in addressing the patient-specific morphological deviations and functional muscle recruitment required for personalized outcomes [13, 14].

Despite these advances, the field still lacks a generalizable, patient-specific computational framework that unifies individual functional inputs, realistic joint contact and soft-tissue mechanics, and subject-specific morphology to produce clinically interpretable metrics such as joint contact forces and intra-articular stress distributions. This study aims to develop and present a data-driven computational framework for TMJ biomechanics that is oriented toward clinical translation and can accommodate diverse clinical scenarios.

To realize this goal, we developed a pipeline comprising (i) a function assessment system that acquires patient-specific functional and morphological data and (ii) a combined multibody dynamics and finite-element (MBD-FE) modeling architecture. This pipeline evaluates changes in functional parameters and mandibular and TMJ morphology, computes joint contact forces, and estimates the magnitude and distribution of stress within the TMJ discs. We illustrate the pipeline’s capabilities with a use case in orthognathic surgery, specifically Le Fort I osteotomy for maxilla repositioning and bilateral sagittal split osteotomy (BSSO) for mandible lengthening or shortening [15, 16]. These procedures involve repositioning the upper or lower jaws to correct skeletal malocclusion and improve facial aesthetics. By applying the pipeline to pre-op and post-op states, we demonstrate one application among several potential clinical uses. Craniofacial skeletal deformities that lead to these surgeries, most commonly skeletal Class II (relative mandibular retrusion) and Class III (relative mandibular prognathism or maxillary deficiency), can alter occlusion, condylar position, and masticatory muscle vectors, thereby changing TMJ loading and possibly causing TMD symptoms (see Online Resource 1 for a visualization of the skeletal classes in the subjects of the study). Since surgical repositioning directly modifies these determinants of load, a pre-/post-operative comparison offers a clinically relevant, controlled test of the framework.

## Materials and Methods

The study incorporated three subjects, selected as representative cases from a larger cohort [17], each representing a distinct skeletal class: one Class I volunteer (normal anteroposterior jaw relationship), one Class II patient (mandibular retrusion relative to the maxilla), and one Class III patient (mandibular prognathism and maxillary deficiency) [18]. The Class II patient underwent BSSO mandibular advancement surgery, while the Class III patient underwent a combined Le Fort I and BSSO mandibular setback surgery. The study was conducted in accordance with ethical guidelines and approved by the Institutional Review Board. Patients underwent functional assessments of the masticatory system, which included acquiring a cone beam computed tomography (CBCT) scan of the entire skull, recording electromyography (EMG) signals from the temporalis and masseter muscles during a unilateral clenching task, and recording the bite force [17, 19]. The Class I subject completed the function assessment once, while the Class II and Class III patients underwent assessment twice, before and 12 months after the surgery. Both surgical patients were asymptomatic and pain-free at the pre-op and post-op states. The data gathered during these assessments was then used to develop the MBD-FE models and evaluate morphology changes.

### Function Assessment System

**Fig. 1:**
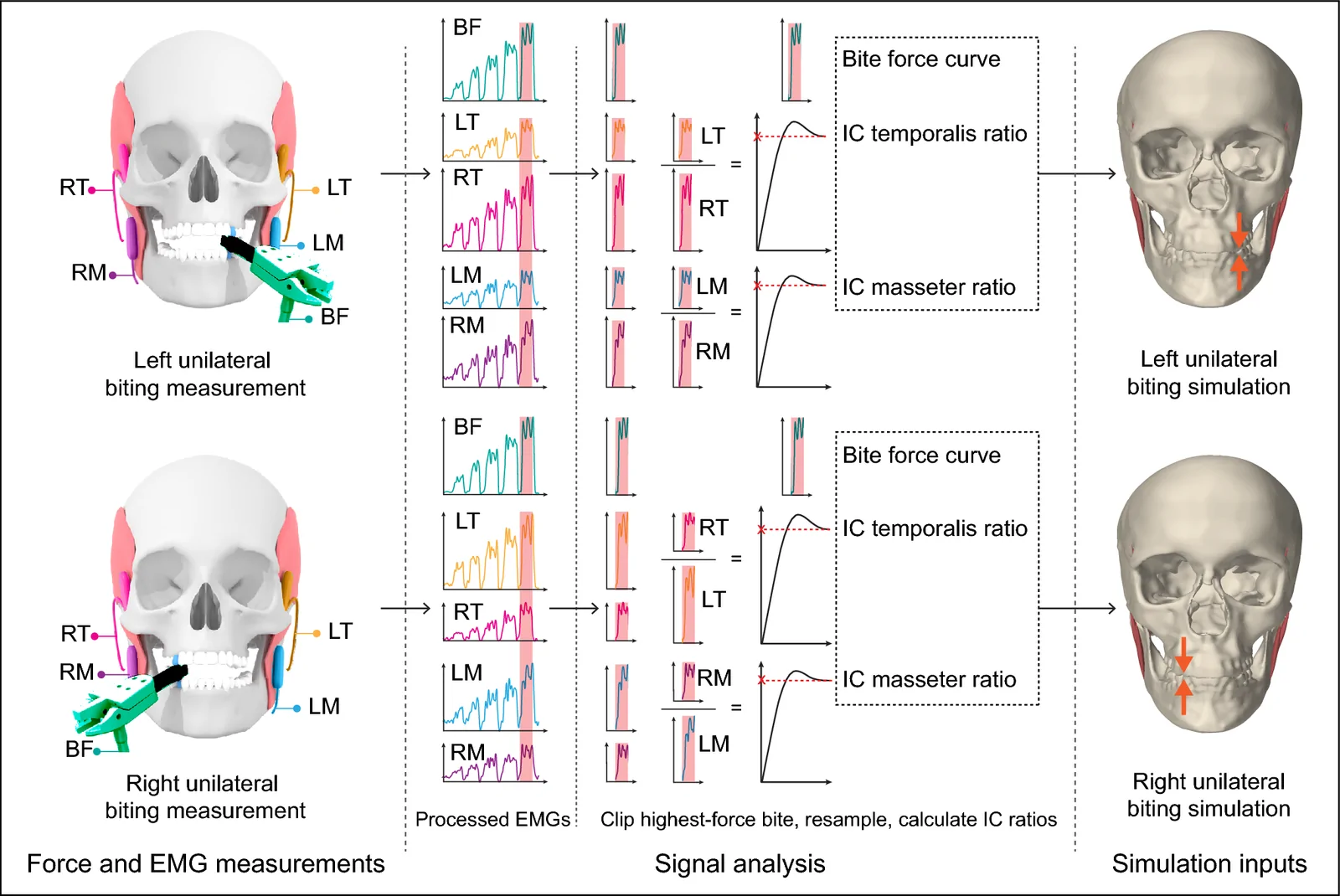
Schematic for data gathering by the function assessment system and preparation steps used for model inputs. *BF* biting force, *LT* left temporalis, *RT* right temporalis, *LM* left masseter, *RM* right masseter

All CBCT scans were acquired at an isotropic voxel size of 0.4 mm (pixel spacing: 0.4 mm × 0.4 mm, slice thickness: 0.4 mm). On the same day as the CBCT acquisition, four SX230 EMG sensors (Biometrics, Newport, UK) were placed on the patient's left and right masseter and temporalis muscles. Additionally, a custom-designed force sensor, developed to measure static bite force at a nearly closed mouth position, was placed between the first premolars on both sides in the same session. Measurements followed a fixed order: left side first, then right; both sides were measured for every participant. The patient performed unilateral bite tasks, each consisting of five consecutive static bites at target force levels of 10 N, 20 N, 30 N, 40 N, and 50 N to the best of their ability [19]. Visual force-level indicators guided the patient during each task. The bite force and EMG signals were recorded at a sampling frequency of 2000 Hz. The EMG signals were detrended, rectified, and low-pass filtered (4th-order Butterworth, 6 Hz cutoff) [20], then normalized based on their baseline value during a 20 N bite [21]. The bite force signal was likewise detrended and low-pass filtered using the same parameters. The force curve corresponding to each patient’s highest force bite level was subsequently clipped and resampled to a duration of 0.25 s for use as an input to the simulation. The corresponding EMG signals were also clipped and resampled (*t* = 0.25 s) and used to calculate a single ipsilateral-to-contralateral (IC) muscle-activation ratio for the left and right temporalis and masseter pairs, inspired by previous studies [2, 22], and served as another input to the MBD-FE model (the preparation of bite force and calculation of task-specific IC ratios for usage as model inputs are illustrated schematically in Fig. 1).

### MBD-FE Model

**Fig. 2:**
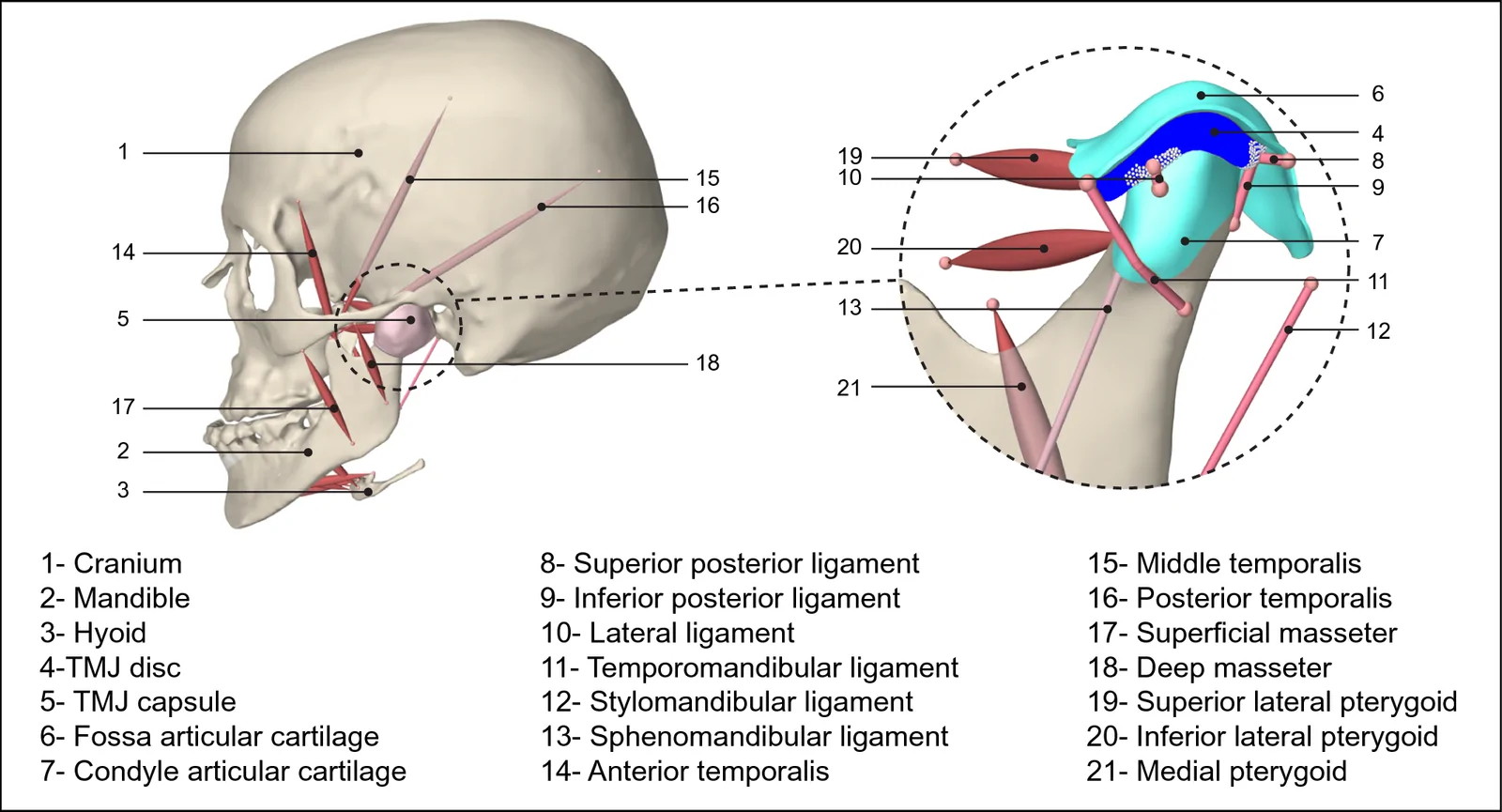
Lateral view of the MBD-FE model developed for the Class III patient in pre-op state, including the detailed view of the left TMJ (submental muscles are not labeled, and the anterior and medial ligaments connecting the TMJ disc to the condyle are not shown)

The MBD-FE models were developed in ArtiSynth, an open-source 3D biomechanical modeling toolkit [23, 24], based on the model presented by Sagl et al. [13] as follows: Cranium, mandible, and hyoid bone were manually segmented out from the CBCT scans acquired as part of the function assessment. All the bones have been modeled as rigid bodies, with the cranium and hyoid kept static and the mandible allowed to move. TMJ discs were defined as FE bodies whose morphology was determined by the shapes of the condylar head of the mandible and the glenoid fossa of the temporal bone [12], and their behavior was modeled as a hyperelastic material [13]. TMJ capsules were defined as FE bodies connecting the boundary of the glenoid fossa to the condylar neck, with an average thickness of 1.97 mm [25] and a hyperelastic material behavior [26] (Table 1). Articular cartilages were simulated as elastic foundations (EFs), a simplified material model to effectively replicate the behavior of the layers covering the surface of the condyle and fossa without simulating them as FE bodies [13]. Seven ligaments in each TMJ were modeled as cables, meaning they only exhibit stiffness when they are under tension: the anterior, medial, and lateral ligaments connecting the disc to the condyle; the posterior ligament, which has two branches connecting the disc to the condyle and the fossa; and the lateral temporomandibular, sphenomandibular, and stylomandibular ligaments connecting the cranium to the mandible. Additionally, the ligaments connecting the disc to the condyle and fossa do not penetrate the condyle and wrap around it. Muscles included in the model were simulated as twelve Hill-type point-to-point elements on each side of the skull [13]. Of these elements, only six on each side get activated in simulating the unilateral biting: the anterior, middle, and posterior temporalis; the superficial and deep masseter; and the medial pterygoid muscle [13] (Fig. 2; Table 1). The attachment sites for muscles and ligaments were identified manually on the segmented CT-based bone geometries. The anatomical locations were determined based on the reference model by Sagl et al. [13] and cross-verified against the Complete Anatomy 3D platform (3D4Medical, Elsevier, Dublin, Ireland) to ensure accuracy of the attachment sites. Furthermore, the complete set of 3D Cartesian coordinates for the muscle and ligament attachment sites for all three subjects is provided in Online Resource 2.

**Table 1:** Model components and their descriptions

Model components	Component type	Mathematical model	Description
Cranium, mandible, and hyoid	Rigid body	Non-deformable	Mass and inertial properties extracted from Sagl et al. [13]
TMJ discs	FE body	Mooney-Rivlin hyperelastic material behavior	Comprised of approximately 30000 first-order tetrahedral elements (edge length ~ 0.3 mm, satisfying mesh convergence criteria in Sagl et al. [13]) with ${C}_{1}$=0.9 MPa and ${C}_{2}$=0.0009 MPa [13] in the following strain energy density function: $W\left({I}_{1},{I}_{2}\right)={C}_{1}\left({I}_{1}-3\right)+{C}_{2}({I}_{2}-3)$
TMJ capsules	FE body	Neo-Hookean hyperelastic material behavior	Comprised of approximately 9000 first-order tetrahedral elements with ${C}_{1}$=1.44 MPa [26] in the following strain energy density function: $W\left({I}_{1}\right)={C}_{1}({I}_{1}-3)$
Articular cartilages	EF body	Elastic foundation behavior	With cartilage thickness h=0.4 mm, elastic modulus E=2.7 MPa, and Poisson’s ratio ν=0.49 [13] in the following formula for contact pressure between the articular cartilage and TMJ disc as a function of penetration d: $p\left(d\right)=K\mathrm{ln}\left(1-\frac{d}{h}\right),$ $K\equiv \frac{-(1-\nu )E}{(1+\nu )(1-2\nu )}$
TMJ ligaments	Elastic cable	Elastic cable behavior with specified slack length	The elongation stiffness of 250 N/unit strain [13, 27], with slack lengths of 4 mm, 1.9 mm, 1.9 mm, 7.5 mm, 4 mm, 0 mm, and 0 mm for the anterior, medial, lateral, posterior, lateral temporomandibular, sphenomandibular, and stylomandibular ligaments, respectively. Slack lengths represent the length added to the initial ligament lengths. For ligament attachment sites, see Online Resource 2.
Muscles	point-to-point muscle	Hill-type muscle behavior [28]	For model parameters, length-force behaviors extracted from Sagl et al. [13], and muscle attachment sites, see Online Resource 2

The developed patient-specific models were then used to simulate unilateral biting. Each biting was simulated by defining a bilateral planar constraint, restricting the motion of a rigid body to a defined plane, allowing two translational and one rotational degree of freedom, while preventing out-of-plane movements [29]. This approach provided a computationally efficient method for simulating contact. The plane of motion was defined by three anatomical landmarks: the tip of the lower left and right first premolars and the lower incisor to closely replicate the unilateral biting task [30]. The resampled unilateral bite force curve was applied at either the left or right first premolar, depending on the simulated task, and oriented normal to the planar connector, setting the target for ArtiSynth’s forward-dynamics force tracking. The muscle activations required to generate the targeted force, along with the internal forces and stresses in the joint, were then predicted, with IC ratios (defined in Fig. 1) enforced for the temporalis and masseter muscle strands.

To compare stress results for TMJ discs across different patients and between pre-op and post-op states, we applied a normalization method to account for variations in biting force and joint geometry observed during function assessments. A “scaling stress” was defined by dividing the unilateral bite force by the condylar area, approximated as the product of the condylar major and minor axes. The reported values are presented as ratios of the simulated von Mises stress to this scaling stress, enabling consistent comparisons regardless of variations in loading and geometry.

### Forward-Dynamics Force Tracking

Following the theoretical framework detailed in Sagl et al. [31], we used the tracking simulation capabilities of ArtiSynth to compute the muscle activations required to generate the measured unilateral bite force by imposing two measured IC ratios for the temporalis and masseter muscles. Unlike inverse dynamics, which calculates forces from a pre-defined motion, this framework integrates the equations of motion forward in time. At each simulation time step, the solver optimizes the muscle activations to drive the forward simulation toward the target force, ensuring that the resulting kinematics and forces satisfy the complex physical constraints of the model (e.g., articular contact and passive tissue compliance). In summary, the force objective function ${\Phi }_{c}$ was used to minimize the difference between a target biting force, $\overline{{\boldsymbol{c}} }$ and the unilateral biting force generated by the muscle activations represented by the term ${{\boldsymbol{H}}}_{{\boldsymbol{c}}}{\boldsymbol{a}}$ in the following equation [31]:

1 $${\Phi }_{c}\left({\boldsymbol{a}}\right)=\frac{1}{2}{w}_{c}\Vert \overline{{\boldsymbol{c}} }-{{\boldsymbol{H}}}_{{\boldsymbol{c}}}{\boldsymbol{a}}\Vert$$

In Eq. (1), ${{\boldsymbol{H}}}_{{\boldsymbol{c}}}$ is the force excitation response matrix where the IC ratios are imposed. Specifically, the columns corresponding to the left and right temporalis and masseter muscle pairs are linearly combined according to the measured IC ratio, effectively reducing the paired muscles to a single activation variable in the optimization. Further, vector ${\boldsymbol{a}}$ is the vector storing the activation signals for all of the muscles involved in achieving the desired unilateral biting task, and ${w}_{c}$ is the weighting assigned to the force objective. Our system is underdefined because the number of muscles needed to perform the task is fewer than the number of muscles available. Consequently, an ${l}^{2}$ regularization term in the form of $\frac{1}{2}{w}_{a}{{\boldsymbol{a}}}^{T}{\boldsymbol{a}}$, which is essentially a weighted square of the excitation values, is added to the objective function to overcome this issue. Additionally, in order to prevent the solution from oscillating between two possible muscle-activation states, a weighted damping term in the form of $\frac{1}{2}{w}_{d}{\Vert {{\boldsymbol{a}}}_{i-1}-{\boldsymbol{a}}\Vert }^{2}$ is included, which serves as a way of minimizing the time derivative of the calculated activations. Ultimately, the optimization objective solved for calculating muscle activations is [31]:

2 $$\mathop {\min }\limits_{\boldsymbol{a}} \frac{1}{2}{w}_{c}\Vert \overline{{\boldsymbol{c}} }-{{\boldsymbol{H}}}_{{\boldsymbol{c}}}{\boldsymbol{a}}\Vert + \frac{1}{2}w_{a} {\boldsymbol{a}}^{T} {\boldsymbol{a}} + \frac{1}{2}{w}_{d}{\Vert {{\boldsymbol{a}}}_{i-1}-{\boldsymbol{a}}\Vert }^{2};{\mathrm{subject}}\;{\mathrm{to}}\;0 \le a \le 1$$

Typically, ${w}_{d}<{w}_{a}\ll {w}_{c}$ [32], and the values used in our simulation are 0.00001, 0.0025, and 1 for ${w}_{d}$, ${w}_{a}$, and ${w}_{c}$, respectively.

### Force Tracking Validation

Although our MBD-FE models were built based on the validated work of Sagl et al. [13], the application of ArtiSynth’s force-tracking capabilities to the patient-specific simulation of masticatory tasks required further investigation. Therefore, we analyzed the effect of the mandible length on the joint contact force for the left unilateral biting simulation of the Class I subject model. To achieve mandible length variation, the average distance from the left and right condyle tops to the menton was adjusted in 5 mm increments across a 105-135 mm range. Attachment points for muscles, ligaments, and anatomical landmarks were then translated along the anterior-posterior axis in proportion to their original distance from the condyle tops to preserve relative geometry. We used this manipulation as a mechanical sanity check. The joint contact force normalized to bite force is expected to vary approximately linearly with the ratio of mandible length to the moment arm of the resultant-muscle forces [19]. We therefore examined whether ArtiSynth’s force-tracking reproduces this relationship in a Class I subject-specific model whose baseline mandibular length (condyle-top to menton = 117.8 mm) lies within published adult ranges [19, 33, 34]. The sweep from 105 to 135 mm was chosen to include the range of reported values [19, 33].

### Morphological Assessments

Morphological measurements, including condylar areas and the distances between the first premolars and condyle tops, were taken as part of TMJ’s biomechanical assessment by a trained observer using established landmarks from previous studies [19, 33]. The measurements, including ipsilateral and contralateral biting arms and condylar areas, were conducted by calculating the distance between anatomic landmarks on 3D geometries segmented from each case's CBCT scan. The landmark selection process was performed three times, and the resulting coordinates were averaged to minimize selection error. To quantify intra-observer variability, the mean radial error (MRE) was calculated for the landmark selection process. The MRE, defined as the average Euclidean deviation of each individual selection from the centroid of the three iterations, was 0.67 mm across all cases, indicating high reproducibility of the landmark placement process.

As another part of TMJ’s biomechanical assessment, and given that stress distribution in the TMJ disc is highly influenced by its contact morphology with adjacent bony structures, two morphological analyses were conducted: joint gap and congruency. The joint gap at each point on the articular surfaces was calculated as the minimum distance of that point to all the points on the opposing surface, using a triangulated mesh with a 0.3 mm average length. Congruency at each point, on the other hand, was evaluated using an equivalent plane and curved surface system in the vicinity of each point [35]. This system represents the curvature difference between the two original articular surfaces at that point. In this method, the principal curvatures of an equivalent surface ${S}_{e}$, ${k}_{max}^{e}$ and ${k}_{min}^{e}$, are calculated by the following equation:

3 $$k_{\max }^{e} , k_{\min }^{e} \; = \;A_{{{\mathrm{s}}_{1} }} \; + \;A_{{{\mathrm{s}}_{2} }} \; \pm \;\sqrt {D_{{{\mathrm{S}}_{1} }}^{2} \; + \;D_{{{\mathrm{S}}_{2} }}^{2} \; - \;2D_{{{\mathrm{S}}_{1} }} D_{{{\mathrm{S}}_{2} }} } ,$$

where $D_{{S_{{\mathrm{i}}} }} \; = \; k_{\min }^{{S_{{\mathrm{i}}} }} \; - \;k_{\max }^{{S_{{\mathrm{i}}} }}$ and $A_{{S_{{\mathrm{i}}} }} \; = \;\frac{1}{2}\left( {k_{\min }^{{S_{{\mathrm{i}}} }} \; + \;k_{\max }^{{S_{{\mathrm{i}}} }} } \right)$ are evaluated based on the principal curvatures of the two original $S_{1}$ and $S_{2}$ surfaces. Using these equivalent principal curvatures, the congruency of the joint at a specific point C is then defined by:

4 $$C\; = \;\sqrt {\frac{{\left( {k_{\max }^{e} } \right)^{2} + \left( {k_{\min }^{e} } \right)^{2} }}{2}}$$

The congruency values determined by equation (4) demonstrate a perfect match of two surfaces at *C* = 0 and a mismatch of the curvature of the two original surfaces with higher values of C.

## Results

The IC ratios calculated from the function assessment measurements and the final bite force values are reported in Table 2. It should be noted that the entire bite force curve was used in the forward-dynamics force tracking; however, only the value at the end of the biting task is reported here, as it serves as the basis for defining the scaling stress and corresponds to the final stress state presented later in the results.

**Table 2:** Measured IC ratios and final bite force values, illustrated in Fig. 1 and used as model inputs

	Left unilateral biting			Right unilateral biting		
	IC temporalis ratio	IC masseter ratio	Final bite force (N)	IC temporalis ratio	IC masseter ratio	Final bite force (N)
Class I	1.52	1.43	51.73	0.71	1.33	48.43
Class II pre-op	1.75	1.69	29.10	0.62	0.34	39.20
Class II post-op	1.07	0.97	56.36	0.61	1.57	54.35
Class III pre-op	0.96	1.34	49.22	0.41	1.07	35.16
Class III post-op	0.69	0.71	72.26	1.37	1.00	61.85

The final bite force values in Table 2 indicate that the Class I subject was able to match the target 50 N bite force-level closely. However, the Class II patient exhibited markedly lower pre-op bite forces, with 29.1 N for left unilateral biting and 39.2 N for right unilateral biting, suggesting a weaker bite force before surgery. Postoperatively, the bite forces increased and approached the 50 N target, pointing to functional improvement. In the Class III patient, a notable increase in bite force was observed postoperatively, suggesting that the patient was more capable of exerting force, even surpassing the 50 N target.

The IC temporalis and masseter ratios also showed notable changes. The Class II patient exhibited a more balanced usage of ipsilateral and contralateral muscles postoperatively, particularly during left unilateral biting. On the other hand, the Class III patient displayed a different pattern, with a decrease in the IC ratios for left unilateral biting but a substantial increase in the IC temporalis ratio for right unilateral biting. These findings indicate that orthognathic surgery influenced muscle coordination with distinct, patient-specific variations.

The morphometric measurements in Table 3 highlight the morphological changes induced by orthognathic surgery. The results indicate that mandibular length was successfully adjusted in both the Class II and Class III patients, bringing the biting arms closer to the values observed in the Class I subject. Additionally, improvements in condylar symmetry were observed, as the size difference between the left and right condylar areas, as well as their deviations from the Class I values, were either reduced or essentially stayed the same postoperatively. Notably, in the Class II patient, the right condyle area (RCA) showed a substantial change post-surgery, reducing the pre-op difference from Class I (56.5 mm2) to a smaller discrepancy (18.0 mm2), reflecting significant condylar remodeling. These measurements collectively suggest that surgery effectively contributed to improving mandibular symmetry.

**Table 3:** Results of morphometric measurements and their definitions

Case	LCA	RCA	LIBA	LCBA	RIBA	RCBA
Class I	119.1	91.0	82.2	103.8	83.6	99.3
Class II pre-op	113.8	147.5	76.2	94.6	74.3	92.6
Difference with Class I	− 5.3	56.5	− 6.0	− 9.2	− 9.3	− 6.7
Class II post-op	112.3	109.0	80.7	100.1	82.2	97.5
Difference with Class I	− 6.8	18.0	− 1.5	− 3.7	− 1.5	− 1.8
Class III pre-op	135.7	150.7	94.7	113.9	94.4	111.1
Difference with Class I	16.6	59.7	12.5	10.1	10.7	11.8
Class III post-op	137.1	141.8	88.7	109.6	90.4	106.3
Difference with Class I	18.0	50.8	6.5	5.9	6.7	7.0

Areas are in mm^2^, and lengths are in mm (see Online Resource 3 for a schematic of the defined morphometric measurements)

*LCA* left condyle area, *RCA* right condyle area, *LIBA* left ipsilateral biting arm, *LCBA* left contralateral biting arm, *RIBA* right ipsilateral biting arm, *RCBA* right contralateral biting arm

**Fig. 3:**
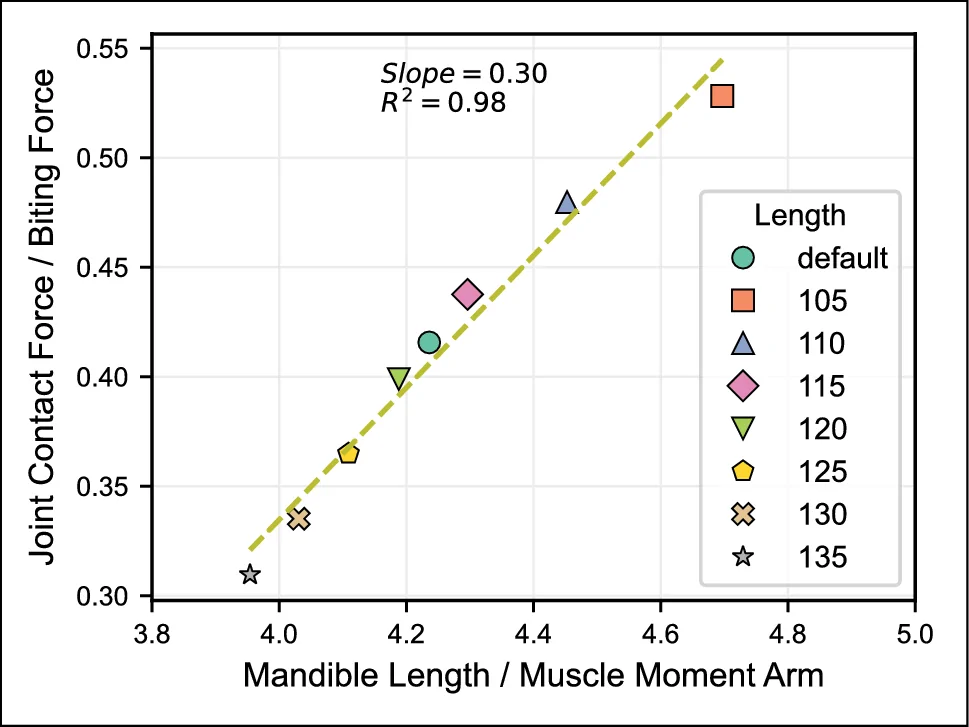
Average bilateral joint contact force (normalized to the bite force) versus the ratio of mandible length to the moment arm of resultant-muscle forces. Each point represents a deterministic simulation at a specified mandible length; the line is a descriptive summary of the expected linear trend

For the Class I subject (left unilateral biting), the normalized joint contact force increased approximately linearly with the ratio of mandible length to the resultant-muscle moment arm (Fig. 3). The default mandible length (condyle-top to menton) was 117.8 mm. A simple linear fit summarized this trend with a slope of 0.30 and ${R}^{2}\,=\,0.98$ across the 105-135 mm range. Each point is a deterministic simulation at a specified geometry.

The predicted joint contact forces [36], obtained by integrating all the forces acting on each disc by the mandible at the final stage of the simulation, are presented in Table 4. The results indicate that the contralateral joint generally experiences a higher contact force during unilateral biting, except for the Class II patient’s pre-op left unilateral biting, where the ipsilateral joint showed a greater contact force. In the post-op evaluation for the Class II patient, this exception was not observed. Additionally, it is notable that the contact force in the right unilateral biting becomes more balanced between left and right joints postoperatively in Class II and Class III patients.

**Table 4:** Simulated joint contact forces as a percentage of respective bite forces

	Left unilateral biting percentage		Right unilateral biting percentage	
	Left joint	Right joint	Left joint	Right joint
Class I	36.0	47.0	55.2	36.0
Class II pre-op	48.1	37.0	92.0	13.6
Class II post-op	39.6	71.3	66.7	55.4
Class III pre-op	55.5	73.9	88.2	42.1
Class III post-op	31.1	85.3	67.6	43.9

**Fig. 4:**
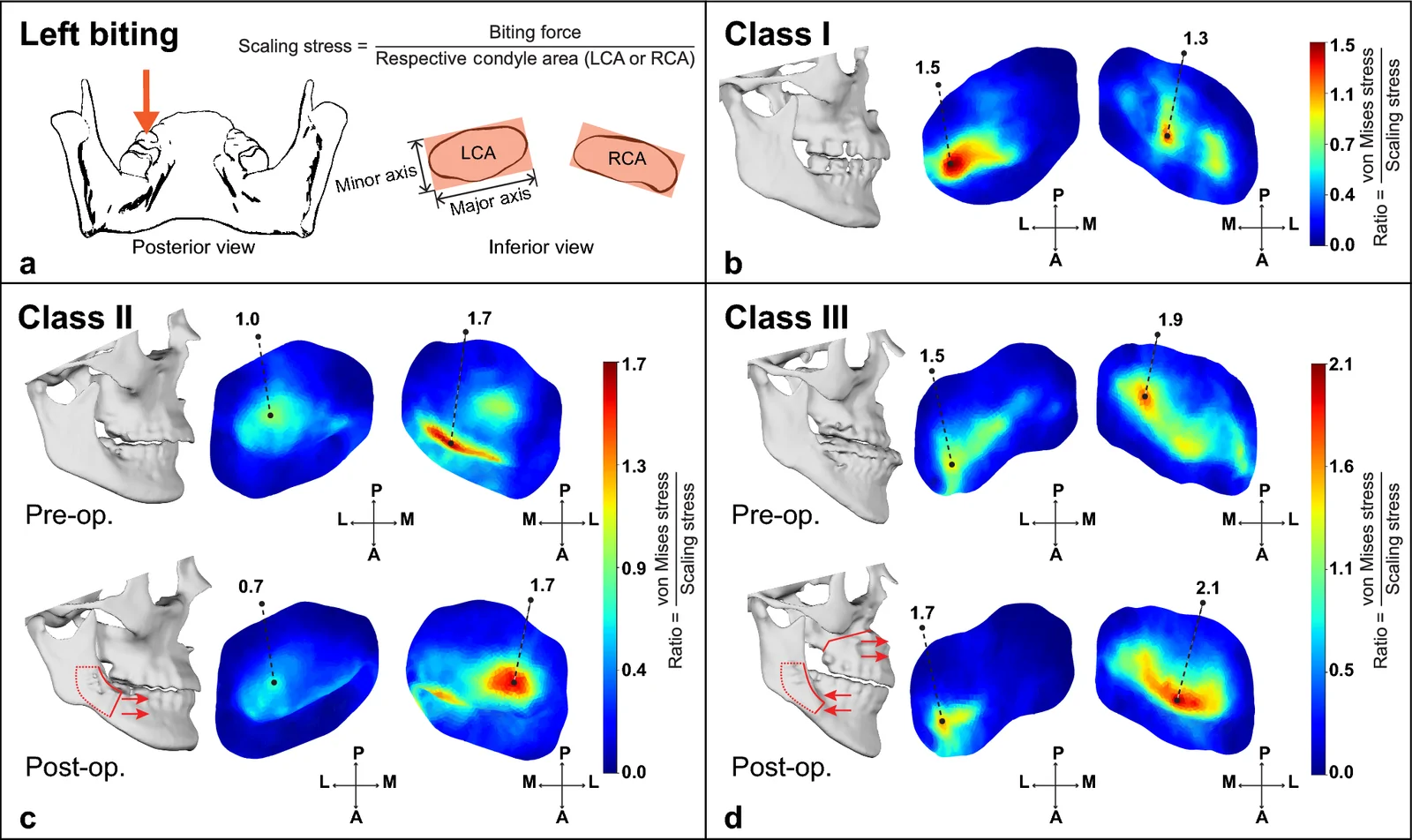
Comparison of the dimensionless stress ratios [von Mises stress (MPa)/Scaling stress (MPa)] for different skeletal classes and pre/post-op states. **a** Left unilateral biting and definition of scaling stress, **b** Class I, **c** Class II pre-op and post-op, and **d** Class III pre-op and post-op. Inferior views of the discs are shown

**Fig. 5:**
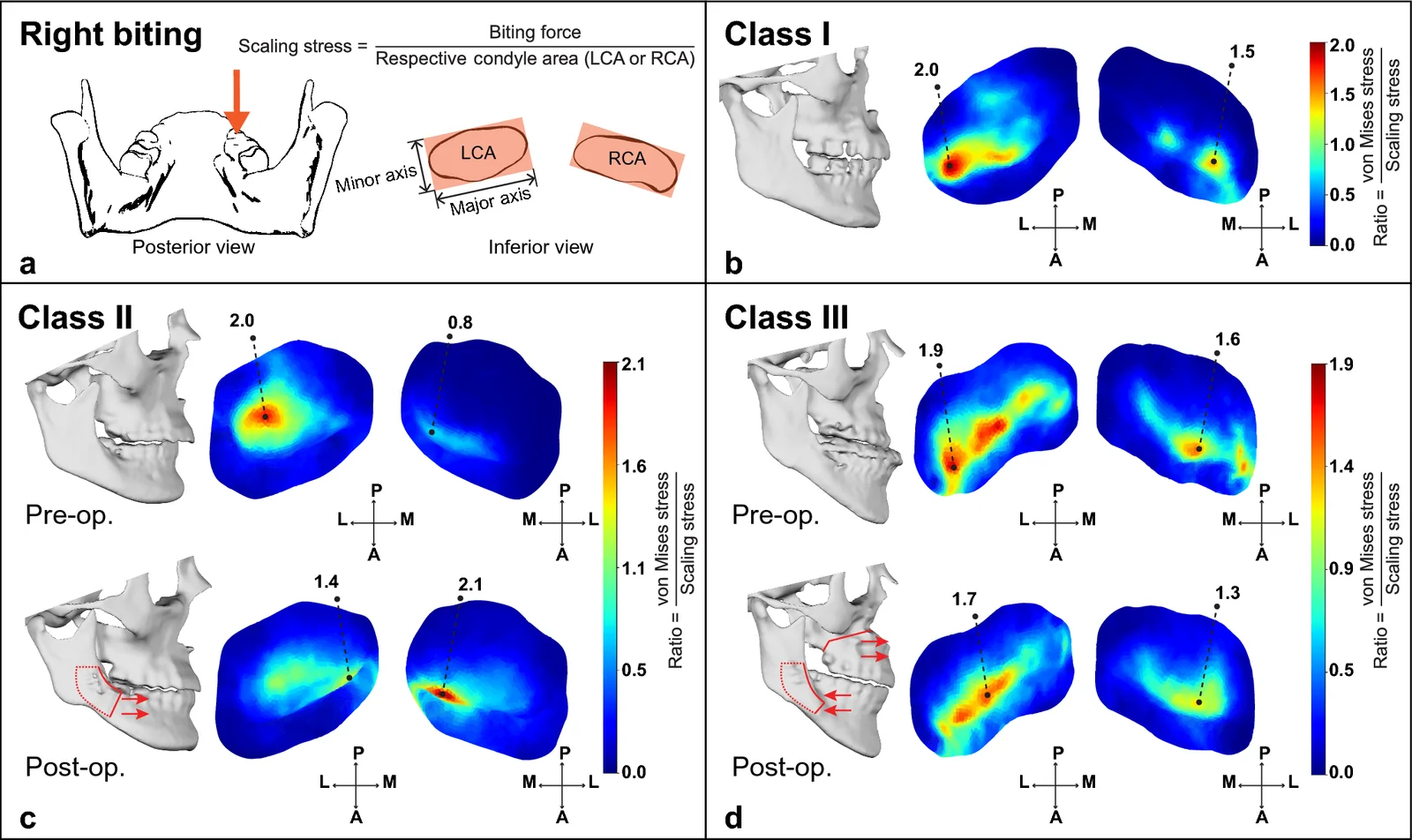
Comparison of the dimensionless stress ratios [von Mises stress (MPa)/Scaling stress (MPa)] for different skeletal classes and pre/post-op states. **a** Right unilateral biting and definition of scaling stress, **b** Class I, **c** Class II pre-op and post-op, and **d** Class III pre-op and post-op. Inferior views of the discs are shown

The scaling stresses used for TMJ disc stress comparisons are listed in Table 5. Stress distribution results for the TMJ discs at the final stage of the left and right unilateral biting tasks are shown in Figs. 4 and 5, respectively. The results in Fig. 4 indicate that the contralateral disc generally experiences higher maximum stresses, except in the Class I subject, where the peak stress ratio, although lower in magnitude compared to the peak stress ratios in the Class II and Class III patients, occurs in the ipsilateral disc. Additionally, in the left TMJ disc (ipsilateral disc for left unilateral biting), the maximum stress is located on the lateral side in Class I and Class III pre-op and post-op states. The maximum stress ratio value has stayed the same for the Class II patient and increased for the Class III patient postoperatively.

**Table 5:** Scaling stress in MPa, defined as respective bite forces divided by condylar areas and used in TMJ disc stress comparison figures (Fig. 4, 5)

	Left unilateral biting		Right unilateral biting	
	Left disc	Right disc	Left disc	Right disc
Class I	0.43	0.57	0.41	0.53
Class II pre-op	0.26	0.20	0.34	0.27
Class II post-op	0.50	0.52	0.48	0.50
Class III pre-op	0.36	0.33	0.26	0.23
Class III post-op	0.53	0.51	0.45	0.44

In Fig. 5 for the right unilateral biting, the contralateral disc generally experiences higher maximum stresses, similar to the results in Fig. 4. An exception is observed in the post-op state of the Class II patient, where a highly localized stress appeared on the medial side of the ipsilateral disc. Notably, after the surgery, the maximum stress ratio shifted from the contralateral disc to the ipsilateral disc in the Class II patient and decreased in the Class III patient.

**Fig. 6:**
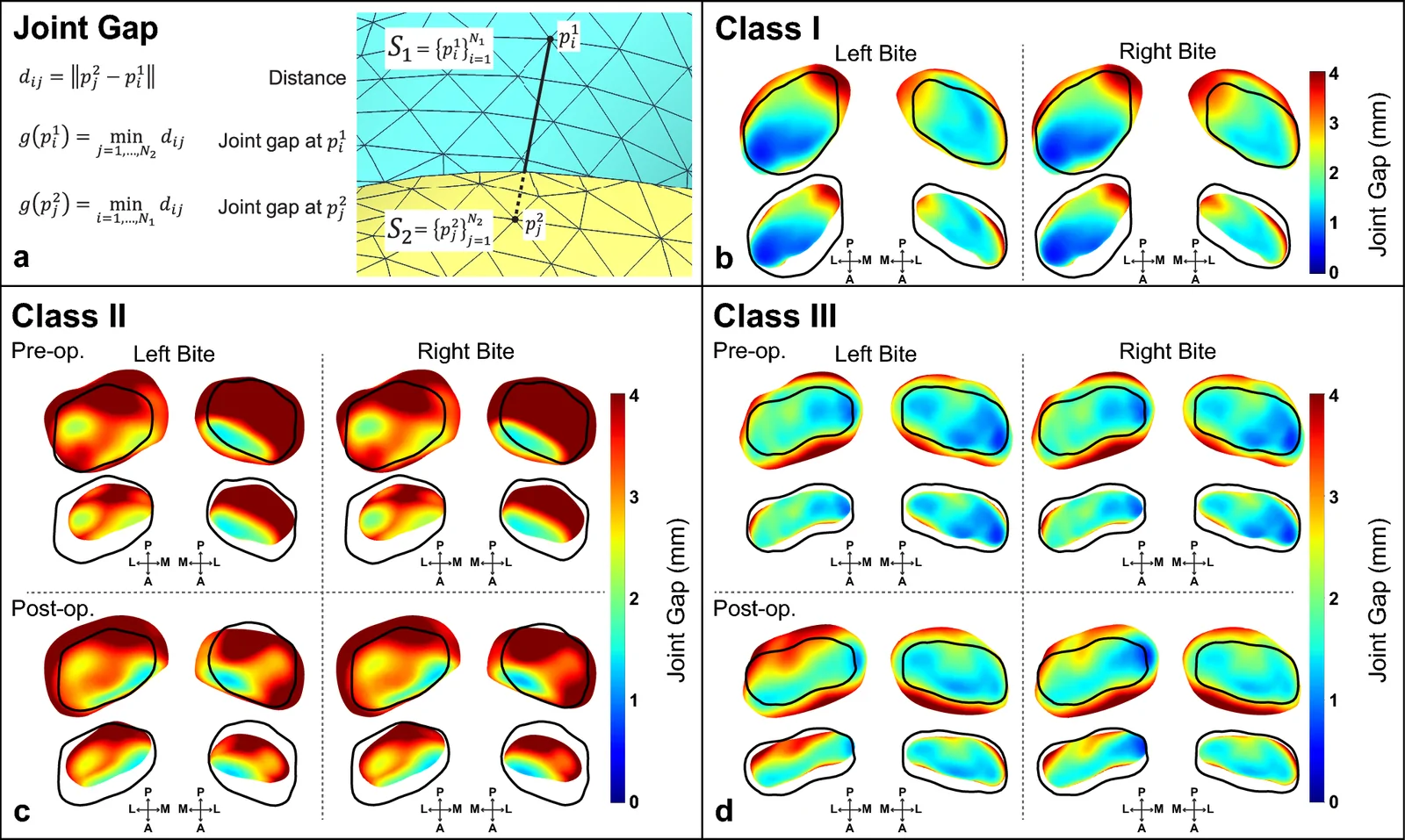
Comparison of the joint gap for different skeletal classes, left and right bite, and pre/post-op states**. a** Joint gap definition, **b** Class I, **c** Class II pre-op and post-op, and **d** Class III pre-op and post-op. Inferior views of the fossae (on the top) and condyles (on the bottom) are shown in the **b**–**d** subfigures. Outlines of the discs are shown on both the fossae and condyles to demonstrate their relative position

**Fig. 7:**
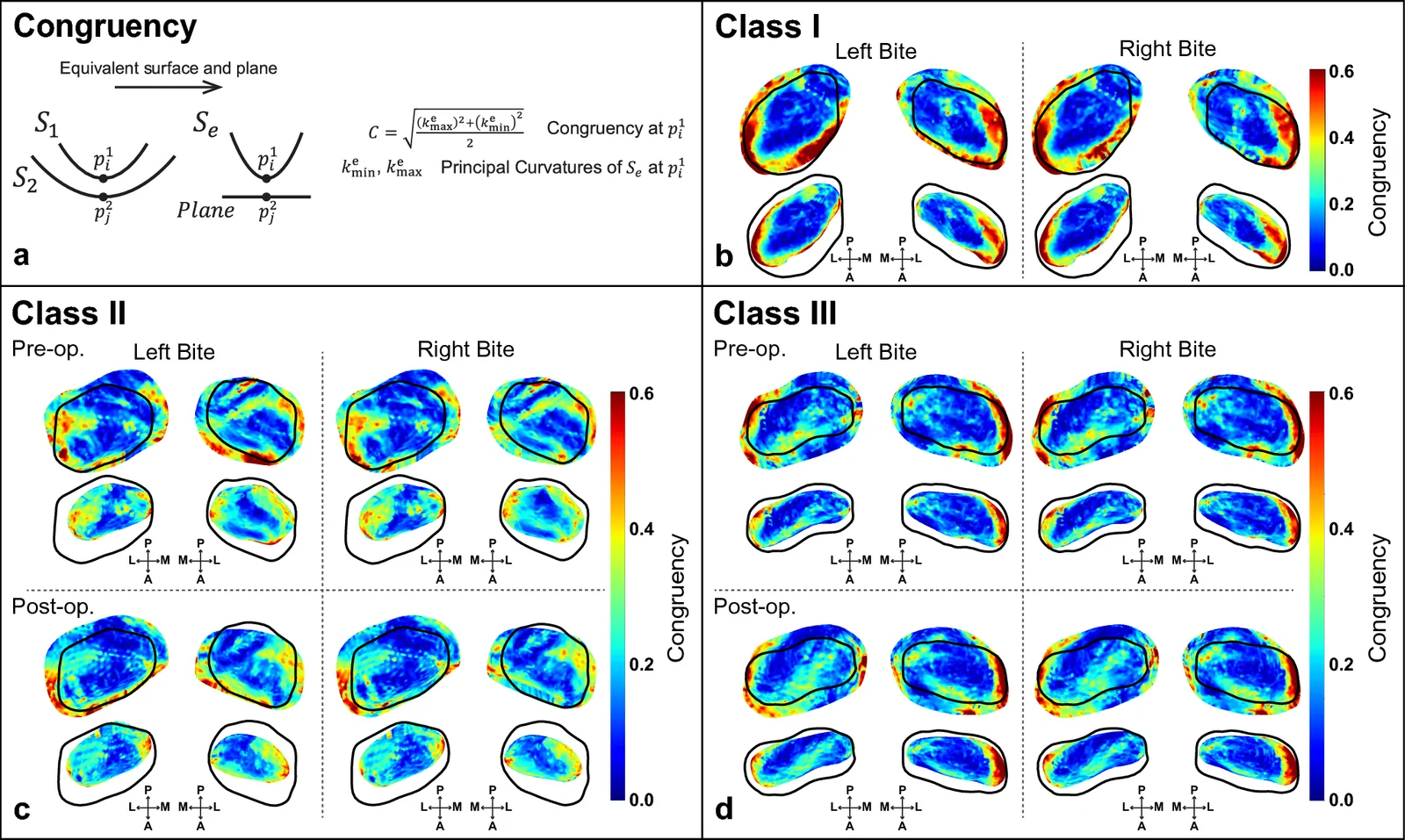
Comparison of the joint congruency for different skeletal classes, left and right bite, and pre/post-op states**. a** Joint congruency definition, **b** Class I, **c** Class II pre-op and post-op, and **d** Class III pre-op and post-op. Inferior views of the fossae (on the top) and condyles (on the bottom) are shown in the **b**, **c**, and **d** subfigures. Outlines of the discs are shown on both the fossae and condyles to demonstrate their relative position

The results of the joint gap and joint congruency analyses are shown in Figs. 6 and 7, respectively. Outlines of the TMJ discs are superimposed on the fossae and condyles to illustrate their relative position. The changes in both the magnitude and patterns of joint gap and congruency following orthognathic surgery are evident. For example, the Class I and Class III patients exhibit relatively small joint gaps compared to the Class II patient. However, orthognathic surgery reduced the joint gaps in the Class II patient, bringing them closer to those of the other two patients. Additionally, the congruency patterns, when superimposed on the discs, indicate a strong correlation with the observed stress patterns in Figs. 4 and 5, particularly in regions where the joint gap is relatively small. A case in point would be the concentrated stress area on the medial side of the right disc in the right biting post-op Class II case (Fig. 5), which corresponds to the smallest local joint gap and a relatively incongruent location, demonstrated in Figs. 6 and 7, respectively.

## Discussion

In this study, we developed and demonstrated a patient-specific MBD-FE pipeline that integrates patient morphology and individualized functional inputs (muscle-usage ratios and biting tasks) to estimate clinically meaningful quantities, including joint contact forces and intra-articular stress distributions, before and after orthognathic surgery.

The functional assessments showed that orthognathic surgery was associated with improved bite force generation and shifts in muscle-activation ratios (Table 2). Our measurements echo prior work showing that ipsilateral/contralateral muscle recruitment during unilateral loading is highly individualized [37]. It is critical to use patient-specific values in simulation because joint contact forces in the MBD-FE are highly sensitive to these input muscle ratios. This sensitivity is exemplified by the Class II patient, where a post-op reduction in left-biting IC ratios (Table 2) coincided with a shift from ipsilateral-to-contralateral joint force dominance (Table 4).

The morphological measurements detailed in Table 3 indicated adaptations in condylar size, most notably in the right condylar area of the Class II and Class III patients, which corresponds to an increase in the joint volume bounded between the glenoid fossa and condyle. This pattern aligns with reported post-op adaptations involving both condylar remodeling and changes in the glenoid fossa [38–40]. In particular, the observed joint space increase is consistent with Kim et al. [39], and condylar volume reduction aligns with van Luijn et al. [38], supporting the view that TMJ morphology adapts following orthognathic intervention. However, a dedicated morphological analysis on a larger longitudinal cohort is required to quantify the extent of these adaptations and their correlation with altered TMJ biomechanics.

Our force-tracking validation results reproduce the linear correlation between normalized joint contact force and the ratio of mandible length to the moment arm of resultant-muscle forces reported by She et al., who found a slope of 0.32 across their population [19]. Our analysis yielded a value of 0.30, providing model-level evidence that ArtiSynth’s force-tracking respects known mechanical scaling relationships in a plausible adult geometry and supports the application to patient-specific simulations without making population-level claims.

Consistent with prior findings, contralateral joint forces exceeded ipsilateral joint forces during unilateral loading (Table 4) [19, 29, 41, 42]. Reported magnitudes of normalized joint forces vary widely across the literature, from values near 0.10 of the applied unilateral loads in Woodford et al. [43] to 0.39-0.93 in de Zee et al. [29] and 0.52-0.94 in She et al. [19], reflecting differences in subject morphology, modeling techniques, and task definition. Given these sources of variability and the patient-specific nature of TMJ mechanics, our predictions fall within published ranges and reinforce the value of individualized assessments.

With respect to stress distributions, our simulations indicate that local stress concentrations (Figs. 4, 5) tend to colocalize with regions of small joint gaps (Fig. 6) and poor articular congruency (Fig. 7) consistent with the notion that disc load distribution depends on condyle-fossa articular interactions [22]. The ranges of stress magnitudes align with similar modeling studies [10, 44]. Nonetheless, direct cross-study comparisons are limited by differences in modeling methods, applied loads, material properties, constitutive laws, and individual morphologies.

Taken together, these results show that orthognathic surgery can substantially alter TMJ biomechanics, but the direction and magnitude of change are not governed by a single factor (e.g., mandible length) alone. Instead, post-op changes emerge from the interplay of altered morphology, including condyle size/shape, fossa geometry, mandible length, contact congruency, joint space, and patient-specific muscle recruitment during biting tasks. A key strength of the present pipeline is that it makes these interactions explicit and quantifiable on a per-patient basis.

The primary limitation of this study is its small sample size, which precludes statistical inference and the detection of broader trends. However, we anticipate that the trend of improved biomechanical balance observed here, specifically the post-op adaptation of IC ratios and consequently the joint contact forces, reflects a generalized tendency toward normalized biomechanics. This is supported by prior findings in larger cohorts [10, 11], where orthognathic surgery has been shown to improve TMJ biomechanical parameters, including reductions in asymmetric joint loading. Building on these insights, future research should expand the cohort to enable population-level analyses.

Additional limitations include the modeling simplifications typical of current TMJ simulations, such as the use of point-to-point muscles and ligaments. Regarding soft-tissue constitutive behavior, the articular disc was modeled as an isotropic hyperelastic material. While the disc natively exhibits region-dependent anisotropy [28, 45], the inclusion of such properties without patient-specific data introduces potential inaccuracies. At the same time, the isotropic formulation maintains computational efficiency and robustness for the analysis of skeletal phenotypes. Furthermore, there are uncertainties regarding subject-specific properties; for instance, ligament slack lengths were adopted from a validated reference model [13] rather than being individualized. While accurate slack length definitions are critical for simulating large mandible movements (e.g., maximum mouth opening), their influence is minimal during the static unilateral clenching analyzed in this study, as the ligaments remain mostly slack in the closed mouth position. Future work extending this pipeline to more dynamic tasks should, however, assess the sensitivity of joint mechanics to these subject-specific variations or the tuning of slack lengths, if dynamic calibration data becomes available. Finally, direct validation of the predicted internal forces and stresses against in vivo measurements remains a challenge due to the current impracticality of such measurements in human TMJs. Future research utilizing animal models and instrumented TMJ implants could provide the necessary ground truth data to rigorously validate these computational predictions.

The feasibility of clinical translation is supported by the nature of the data acquisition protocol, which utilizes non-invasive, standard clinical tools (surface EMG, bite force sensors, and CBCT). Clinically, the biomechanical readouts, specifically joint contact forces, muscle recruitment ratios, and stress maps, offer distinct decision support value. For example, identifying stress concentrations could inform modifications to surgical planning to mitigate local overloading. Furthermore, these metrics allow for the identification of patients at risk of tissue degeneration, facilitating personalized management and targeted post-operative rehabilitation.

In summary, the proposed patient-specific MBD-FE pipeline, informed by individualized functional data and morphology, captures tangible changes in TMJ joint forces and disc stress patterns following orthognathic surgery. By linking function, morphology, and internal joint mechanics within a unified framework, it provides a foundation for patient-tailored assessment and, ultimately, for decision support in orthognathic care.

## Supplementary Information

Below is the link to the electronic supplementary material.Supplementary file1 (PDF 1169 kb)

## Data Availability

The CBCT data, MBD-FE models, and analytic codes used in this study are available upon request.
