# Supplementary material for: A Computational Framework for Simulating Patient-Specific TMJ Biomechanics Using a Combined Multibody Dynamics and Finite Element Approach
Source: Ann Biomed Eng. Author manuscript; Available in PMC 2026 Apr 9. (PMC13063518; doi:10.1007/s10439-026-04020-0)

Online Resource 1

Skeletal classes illustrated by the study subjects. **a** Class I volunteer with no skeletal deformity, **b** Class II patient with mandibular retrusion relative to the maxilla, and **c** Class III patient with both mandibular prognathism and maxillary deficiency. The lateral view is shown in all the subfigures. Class II and Class III patients are shown in their pre-op state.

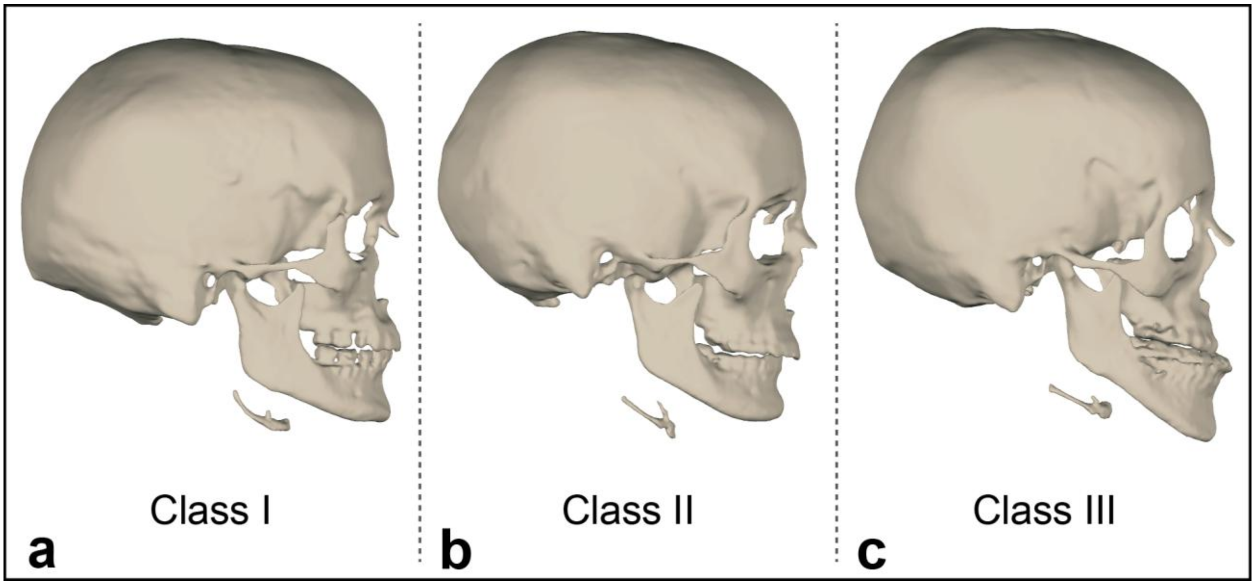

Online Resource 2

Hill-Type Muscle Model Formulation and Parameters, and Muscle and Ligament Attachment Sites

1. Hill-Type Muscle Model Formulation

The masticatory muscles were modeled as point-to-point Hill-type actuators. The total force F^{total} generated by each muscle is calculated as the sum of active contractile force and passive elastic force. Our implementation follows the Hill-type formulation tailored for jaw biomechanics[1]:

$$F^{total} = F^{max} \cdot \left[a \cdot f_{active}(\tilde{l}) + P_{frac} \cdot f_{passive}(l)\right]$$

Where:

- F^{max} is the maximum muscle force (N).
- a is the activation level ($0 \leq a \leq 1$).
- f_{active} is the active force component.
- $\tilde{l}$ is the normalized fiber length.
- P_{frac} is the passive fraction coefficient. This value is set as 0.015[1].
- $f_{passive}$ is the passive elastic component.
- l is the total muscle length, including the tendon.

Maximum Force F^{max} is calculated as the product of the muscle's Physiological Cross-Sectional Area ($PCSA$) and a specific tension constant of 40 N/cm^2[2].

The activation level a is calculated by the Forward-Dynamics Force Tracking simulation.

The active force component f_{active} describes the force generation capacity of the sarcomeres as a function of normalized fiber length $\tilde{l}$. The model utilizes a cosine-based approximation of the force-length curve[1]:

$$f_{active}(\tilde{l}) = \begin{cases} 0.5 \cdot \left(1 + \cos\left(2\pi \cdot \tilde{l}\,\right)\right) & if\ 0.5 < \tilde{l}\ < 1.5 \\ 0 & otherwise \end{cases}$$

The normalized fiber length $\tilde{l}$ is calculated by subtracting the tendon length (L^{tendon}) from the current total muscle length (l) and normalizing by the optimal fiber length (L^{opt}_{fiber})[1]:

$$\tilde{l} = \frac{l - L^{tendon}}{L^{opt}_{fiber}}$$

where:

$$L^{tendon} = L^{opt} \cdot R_{tendon}$$

$$(L^{opt}_{fiber} = L^{opt} \cdot (1 - R_{tendon})$$

L^{opt} is the total muscle length at which active force is maximal, and R_{tendon} is the tendon-to-muscle length ratio. In our implementation, L^{opt} was assigned to be the muscle length in the resting position derived from the subject's geometry[1].

$$L^{opt} = L_{resting\ position}$$

L^{max}, which is the maximum muscle length, was then calculated so that it maintains the optimal-to-maximum length ratio defined in the literature[1].

$$L^{max} = L^{opt} \cdot \frac{L^{max}_{generic}}{L^{opt}_{generic}}$$

The passive elastic component $f_{passive}$ represents the resistance of the muscle tissue to stretching. In our implementation, a linear ramp function was used to approximate the passive curve[1]:

$$f_{passive}(l) = \begin{cases} 0 & if\ l\ \leq L^{opt} \\ \dfrac{l - L^{opt}}{L^{max} - L^{opt}} & if\ L^{opt} < l < L^{max} \\ 1.0 & if\ l \geq L^{max} \end{cases}$$

2. Muscle Model Parameters

The parameters governing the muscle model were assigned according to the following tables (**Table 1**, **Table 2**, **Table 3**, and **Table 4**):

Muscle Name	$PCSA$ (cm^2)	$F^{max}(N)$	$L^{opt}_{generic}(mm)$	$L^{max}_{generic}(mm)$	R_{tendon}
Anterior Temporalis	3.95[2]	158.0[2]	75.5[2]	95.9[2]	0.50[2]
Middle Temporalis	2.39[2]	95.6[2]	65.8[2]	93.4[2]	0.48[2]
Posterior Temporalis	1.89[2]	75.6[2]	77.1[2]	101.1[2]	0.51[2]
Superficial Masseter	4.76[2]	190.4[2]	51.5[2]	66.9[2]	0.46[2]
Deep Masseter	2.04[2]	81.6[2]	29.1[2]	44.9[2]	0.29[2]
Medial Pterygoid	4.37[2]	174.8[2]	40.5[2]	50.6[2]	0.64[2]
Inferior Lateral Pterygoid	1.67[2]	66.9[2]	31.5[1]	41.5[1]	0.00[a][1]
Superior Lateral Pterygoid	0.72[b][1]	28.7[b][1]	27.7[1]	37.7[1]	0.00[a][1]
Anterior Digastric	1.00[2]	40.0[2]	35.1[c][1]	45.1[c][1]	0.00[a][1]
Anterior Mylohyoid	0.89[d][1]	35.4[1]	35.1[c][1]	45.1[c][1]	0.00[a][1]
Posterior Mylohyoid	0.89[d][1]	35.4[1]	35.1[c][1]	45.1[c][1]	0.00[a][1]
Geniohyoid	0.80[3]	32.0[1]	35.1[c][1]	45.1[c][1]	0.00[a][1]

Table 1: Literature-derived parameters of the Hill-type muscle actuators that are constant across subjects

[a] In our implementation, R_{tendon} was assigned to be 0 for these muscles[1].

[b] Modeled as 30% of the total Lateral Pterygoid capacity, with the Inferior head representing the other 70%, derived from Peck et al.[2].

[c] In our implementation, the $L^{opt}_{generic}$ and $L^{max}_{generic}$ for Anterior Digastric, Anterior and Posterior Mylohyoid, and Geniohyoid were assigned to be the same values[1].

[d] The total Mylohyoid ($PCSA = 1.77\ cm^2$[3]) was divided equally between the Anterior and Posterior segments.

Muscle	Subject	L^{opt} (mm)		$L^{max}(mm)$	
		Left	Right	Left	Right
Anterior Temporalis	Class I	69.3	70.7	87.9	89.8
	Class II pre-op	62.3	62.6	79.1	79.5
	Class II post-op	64.7	63.3	82.2	80.3
	Class III pre-op	64.0	61.8	81.2	78.5
	Class III post-op	65.4	59.2	83.1	75.2
Middle Temporalis	Class I	101.5	100.0	144.0	141.9
	Class II pre-op	100.4	105.3	142.4	149.3
	Class II post-op	109.5	105.6	155.4	149.9
	Class III pre-op	102.2	96.2	145.0	136.5
	Class III post-op	103.4	96.4	146.7	136.7
Posterior Temporalis	Class I	125.5	122.5	164.5	160.6
	Class II pre-op	109.0	113.9	142.9	149.3
	Class II post-op	117.7	113.9	154.2	149.4
	Class III pre-op	119.4	114.6	156.5	150.2
	Class III post-op	118.9	114.3	155.9	149.8
Superficial Masseter	Class I	49.5	51.4	64.4	66.8
	Class II pre-op	48.7	51.1	63.3	66.4
	Class II post-op	49.3	45.2	64.1	58.7
	Class III pre-op	41.6	40.2	54.0	52.3
	Class III post-op	45.2	41.5	58.8	54.0
Deep Masseter	Class I	41.6	41.2	64.2	63.5
	Class II pre-op	39.2	41.5	60.4	64.0
	Class II post-op	39.5	36.6	60.9	56.4
	Class III pre-op	35.9	36.1	55.4	55.6
	Class III post-op	39.1	38.6	60.3	59.5

Table 2: Subject-specific muscle length parameters L^{opt} and L^{max} for the Temporalis and Masseter muscles

Muscle	Subject	$L^{opt}\ (mm)$		$L^{max}\ (mm)$	
		Left	Right	Left	Right
Medial Pterygoid	Class I	39.1	41.3	48.8	51.6
	Class II pre-op	40.4	42.5	50.6	53.1
	Class II post-op	38.6	37.3	48.2	46.6
	Class III pre-op	34.7	37.4	43.4	46.8
	Class III post-op	41.1	38.5	51.3	48.2
Inferior Lateral Pterygoid	Class I	37.0	37.4	48.7	49.3
	Class II pre-op	36.0	35.1	47.4	46.2
	Class II post-op	35.8	33.8	47.1	44.6
	Class III pre-op	32.2	34.9	42.4	45.9
	Class III post-op	32.9	35.1	43.4	46.3
Superior Lateral Pterygoid	Class I	35.1	36.1	47.7	49.2
	Class II pre-op	34.4	33.8	46.8	46.1
	Class II post-op	33.9	37.1	46.1	50.4
	Class III pre-op	29.9	33.1	40.7	45.1
	Class III post-op	30.2	32.6	41.1	44.4

Table 3: Subject-specific muscle length parameters L^{opt} and L^{max} for the Pterygoid muscles

Muscle	Subject	L^{opt} (mm)		L^{max} (mm)	
		Left	Right	Left	Right
Anterior Digastric	Class I	44.6	46.2	57.3	59.3
	Class II pre-op	42.0	41.0	53.9	52.7
	Class II post-op	44.2	44.2	56.8	56.8
	Class III pre-op	48.2	47.6	61.9	61.2
	Class III post-op	43.1	43.4	55.4	55.7
Anterior Mylohyoid	Class I	31.3	30.7	40.2	39.4
	Class II pre-op	35.1	33.2	45.1	42.7
	Class II post-op	36.1	34.5	46.4	44.3
	Class III pre-op	32.6	33.6	41.9	43.2
	Class III post-op	27.1	29.3	34.8	37.7
Posterior Mylohyoid	Class I	31.4	30.8	40.3	39.6
	Class II pre-op	33.4	31.0	42.9	39.9
	Class II post-op	33.1	30.0	42.5	38.6
	Class III pre-op	30.8	31.2	39.6	40.1
	Class III post-op	26.7	28.1	34.4	36.2
Geniohyoid	Class I	43.1	43.3	55.4	55.6
	Class II pre-op	42.4	42.1	54.4	54.1
	Class II post-op	45.7	44.9	58.7	57.7
	Class III pre-op	43.8	44.5	56.3	57.2
	Class III post-op	39.3	40.5	50.5	52.1

Table 4: Subject-specific muscle length parameters L^{opt} and L^{max} for the Digastric, Mylohyoid, and Geniohyoid muscles

3. Muscle and Ligament Attachment Sites

The three-dimensional Cartesian coordinates (X, Y, Z) for all the muscle and ligament attachment sites are provided according to the following:

- Class I Volunteer: Muscle attachment sites in **Table 5** and **Fig. 1**. Ligament attachment sites in **Table 10** and **Fig. 6**.
- Class II Patient: Pre-op and post-op muscle attachment sites in **Table 6**, **Fig. 2**, **Table 7**, and **Fig. 3**, respectively. Pre-op and post-op ligament attachment sites in **Table 11**, **Fig. 7**, **Table 12**, and **Fig. 8**, respectively.
- Class III Patient: Pre-op and post-op muscle attachment sites in **Table 8**, **Fig. 4**, **Table 9**, and **Fig. 5**, respectively. Pre-op and post-op ligament attachment sites in **Table 13**, **Fig. 9**, **Table 14**, and **Fig. 10**, respectively.

All coordinates (mm) are defined relative to the subject-specific coordinate system. The coordinate system origin is located on the sagittal plane. The positive X, Y, and Z axes are defined as follows: $+X$ points to the subject left, $+Y$ points posterior, and $+Z$ points Superior.

Muscle	Attachment site 1	$X\ (mm)$	$Y\ (mm)$	$Z\ (mm)$	Attachment site 2	$X\ (mm)$	$Y\ (mm)$	$Z\ (mm)$
L Anterior Temporalis	Cranium	47.8	-61.2	49.8	Mandible	35.1	-40.2	-14.9
R Anterior Temporalis	Cranium	-47.5	-64.8	51.2	Mandible	-38.1	-40.6	-14.6
L Middle Temporalis	Cranium	66.1	12.8	77.9	Mandible	39.1	-46.8	0.3
R Middle Temporalis	Cranium	-64.2	8.9	78.6	Mandible	-41.9	-47.5	-0.9
L Posterior Temporalis	Cranium	55.6	66.8	51.0	Mandible	39.1	-46.8	0.3
R Posterior Temporalis	Cranium	-58.6	61.8	52.0	Mandible	-41.9	-47.5	-0.9
L Superficial Masseter	Cranium	43.7	-56.5	-3.5	Mandible	39.8	-34.6	-47.7
R Superficial Masseter	Cranium	-45.6	-57.7	-2.2	Mandible	-42.9	-34.0	-47.8
L Deep Masseter	Cranium	52.6	-33.8	5.1	Mandible	43.1	-22.9	-34.0
R Deep Masseter	Cranium	-51.9	-33.9	5.0	Mandible	-46.0	-22.4	-34.1
L Medial Pterygoid	Cranium	18.1	-36.8	-8.7	Mandible	37.4	-26.3	-41.0
R Medial Pterygoid	Cranium	-18.5	-37.3	-7.4	Mandible	-39.8	-27.4	-41.4
L Inferior Lateral Pterygoid	Cranium	16.6	-41.0	-2.4	Mandible	42.6	-15.1	-7.3
R Inferior Lateral Pterygoid	Cranium	-18.5	-41.0	-2.1	Mandible	-45.7	-15.6	-5.5
L Superior Lateral Pterygoid	Cranium	16.7	-36.1	4.4	Mandible	42.6	-13.6	-2.9
R Superior Lateral Pterygoid	Cranium	-18.9	-37.0	4.7	Mandible	-47.0	-14.8	0.0
L Anterior Digastric	Mandible	8.4	-75.8	-65.0	Hyoid	12.2	-31.4	-66.5
R Anterior Digastric	Mandible	-10.2	-76.5	-64.3	Hyoid	-13.1	-30.4	-65.6
L Anterior Mylohyoid	Mandible	15.9	-63.6	-56.9	Hyoid	2.4	-39.9	-72.4
R Anterior Mylohyoid	Mandible	-18.0	-63.4	-58.2	Hyoid	-3.4	-40.6	-72.4
L Posterior Mylohyoid	Mandible	19.4	-49.0	-45.4	Hyoid	8.7	-34.4	-71.0
R Posterior Mylohyoid	Mandible	-23.3	-46.6	-45.7	Hyoid	-10.5	-33.4	-70.4
L Geniohyoid	Mandible	3.1	-79.0	-66.6	Hyoid	2.4	-36.1	-70.6
R Geniohyoid	Mandible	-3.8	-79.3	-66.2	Hyoid	-3.8	-36.3	-70.8

Table 5: Muscle attachment sites for the Class I volunteer (L, left; R, right)

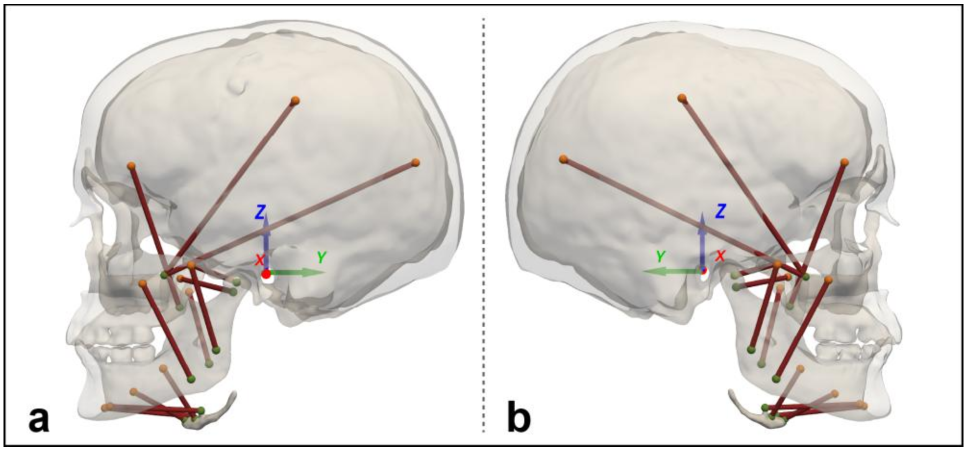

Fig. 1: Visualization of the Class I volunteer muscle attachment sites **(a)** Left side attachment sites and **(b)** Right side attachment sites. The cranium and mandible are rendered transparently for visualization purposes. Orange markers indicate the attachment site 1, and green markers indicate the attachment site 2 in **Table 5**.

Muscle	Attachment site 1	$X\ (mm)$	$Y\ (mm)$	$Z\ (mm)$	Attachment site 2	$X\ (mm)$	$Y\ (mm)$	$Z\ (mm)$
L Anterior Temporalis	Cranium	50.8	-60.4	13.0	Mandible	40.2	-31.7	-41.3
R Anterior Temporalis	Cranium	-47.3	-56.3	17.4	Mandible	-41.8	-31.2	-39.7
L Middle Temporalis	Cranium	55.7	5.4	54.6	Mandible	43.1	-34.1	-36.8
R Middle Temporalis	Cranium	-51.8	9.8	59.1	Mandible	-43.8	-34.1	-36.2
L Posterior Temporalis	Cranium	48.6	58.8	19.9	Mandible	43.1	-34.1	-36.8
R Posterior Temporalis	Cranium	-49.7	62.6	23.7	Mandible	-43.8	-34.1	-36.2
L Superficial Masseter	Cranium	49.6	-43.8	-38.8	Mandible	39.1	-20.8	-80.5
R Superficial Masseter	Cranium	-53.7	-38.4	-32.1	Mandible	-43.3	-17.3	-77.4
L Deep Masseter	Cranium	56.8	-24.5	-34.2	Mandible	41.5	-11.0	-67.7
R Deep Masseter	Cranium	-59.3	-19.6	-27.6	Mandible	-44.7	-7.4	-64.4
L Medial Pterygoid	Cranium	15.3	-25.3	-41.1	Mandible	33.7	-10.8	-74.0
R Medial Pterygoid	Cranium	-17.1	-24.1	-38.9	Mandible	-37.9	-5.7	-71.0
L Inferior Lateral Pterygoid	Cranium	19.0	-33.0	-35.4	Mandible	43.3	-6.6	-39.0
R Inferior Lateral Pterygoid	Cranium	-20.3	-30.8	-33.2	Mandible	-44.9	-6.3	-37.9
L Superior Lateral Pterygoid	Cranium	21.0	-33.6	-54.9	Mandible	42.2	-8.5	-44.9
R Superior Lateral Pterygoid	Cranium	-22.7	-31.5	-49.7	Mandible	-44.1	-6.4	-42.1
L Anterior Digastric	Mandible	9.8	-54.5	-88.9	Hyoid	11.9	-12.8	-93.5
R Anterior Digastric	Mandible	-13.5	-53.5	-87.9	Hyoid	-17.9	-13.0	-92.4
L Anterior Mylohyoid	Mandible	14.1	-45.4	-85.5	Hyoid	-1.1	-21.3	-106.0
R Anterior Mylohyoid	Mandible	-17.5	-44.5	-84.4	Hyoid	-6.0	-21.5	-105.4
L Posterior Mylohyoid	Mandible	19.7	-31.6	-76.3	Hyoid	5.0	-19.5	-103.7
R Posterior Mylohyoid	Mandible	-22.3	-30.9	-75.4	Hyoid	-11.6	-19.4	-102.2
L Geniohyoid	Mandible	0.9	-60.2	-91.4	Hyoid	-1.1	-19.3	-102.2
R Geniohyoid	Mandible	-4.4	-59.7	-91.3	Hyoid	-5.6	-19.0	-101.9

Table 6: Muscle attachment sites for the Class II patient pre-op (L, left; R, right)

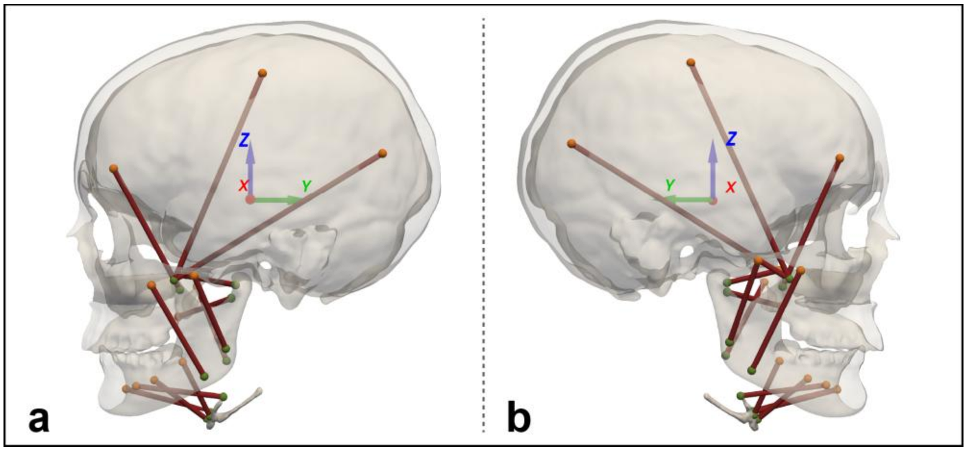

Fig. 2: Visualization of the Class II patient pre-op muscle attachment sites **(a)** Left side attachment sites and **(b)** Right side attachment sites. The cranium and mandible are rendered transparently for visualization purposes. Orange markers indicate the attachment site 1, and green markers indicate the attachment site 2 in **Table 6**.

Muscle	Attachment site 1	$X\ (mm)$	$Y\ (mm)$	$Z\ (mm)$	Attachment site 2	$X\ (mm)$	$Y\ (mm)$	$Z\ (mm)$
L Anterior Temporalis	Cranium	51.7	-58.3	16.5	Mandible	41.7	-32.6	-42.1
R Anterior Temporalis	Cranium	-47.3	-54.5	25.7	Mandible	-43.7	-28.1	-31.6
L Middle Temporalis	Cranium	52.1	12.6	60.1	Mandible	45.0	-34.7	-38.5
R Middle Temporalis	Cranium	-42.3	15.6	68.0	Mandible	-46.2	-30.0	-27.2
L Posterior Temporalis	Cranium	46.3	61.9	28.7	Mandible	45.0	-34.7	-38.5
R Posterior Temporalis	Cranium	-46.2	64.8	36.1	Mandible	-46.2	-30.0	-27.2
L Superficial Masseter	Cranium	50.0	-44.2	-37.7	Mandible	36.4	-19.3	-78.0
R Superficial Masseter	Cranium	-53.3	-40.5	-31.0	Mandible	-44.3	-19.1	-69.8
L Deep Masseter	Cranium	56.4	-24.0	-34.4	Mandible	39.4	-11.2	-67.6
R Deep Masseter	Cranium	-59.2	-21.0	-26.4	Mandible	-45.4	-10.8	-58.7
L Medial Pterygoid	Cranium	15.5	-29.1	-42.9	Mandible	30.8	-9.6	-72.5
R Medial Pterygoid	Cranium	-17.4	-27.1	-41.2	Mandible	-38.4	-11.0	-67.5
L Inferior Lateral Pterygoid	Cranium	19.3	-31.7	-32.9	Mandible	45.3	-8.8	-42.2
R Inferior Lateral Pterygoid	Cranium	-19.3	-25.0	-31.9	Mandible	-45.6	-3.7	-34.2
L Superior Lateral Pterygoid	Cranium	20.5	-33.6	-55.3	Mandible	43.4	-10.5	-45.9
R Superior Lateral Pterygoid	Cranium	-23.0	-31.5	-51.3	Mandible	-44.7	-4.2	-38.7
L Anterior Digastric	Mandible	7.8	-59.4	-89.8	Hyoid	9.4	-15.3	-92.6
R Anterior Digastric	Mandible	-17.7	-58.4	-89.0	Hyoid	-20.2	-14.3	-90.5
L Anterior Mylohyoid	Mandible	11.9	-50.4	-84.9	Hyoid	-4.4	-24.1	-103.6
R Anterior Mylohyoid	Mandible	-21.4	-49.1	-83.2	Hyoid	-8.5	-24.1	-103.1
L Posterior Mylohyoid	Mandible	17.8	-38.0	-77.1	Hyoid	2.1	-22.1	-101.5
R Posterior Mylohyoid	Mandible	-24.6	-35.9	-75.6	Hyoid	-14.8	-21.5	-100.0
L Geniohyoid	Mandible	-1.1	-66.3	-93.0	Hyoid	-4.3	-21.4	-100.5
R Geniohyoid	Mandible	-7.4	-65.6	-92.4	Hyoid	-8.2	-21.4	-100.1

Table 7: Muscle attachment sites for the Class II patient post-op (L, left; R, right)

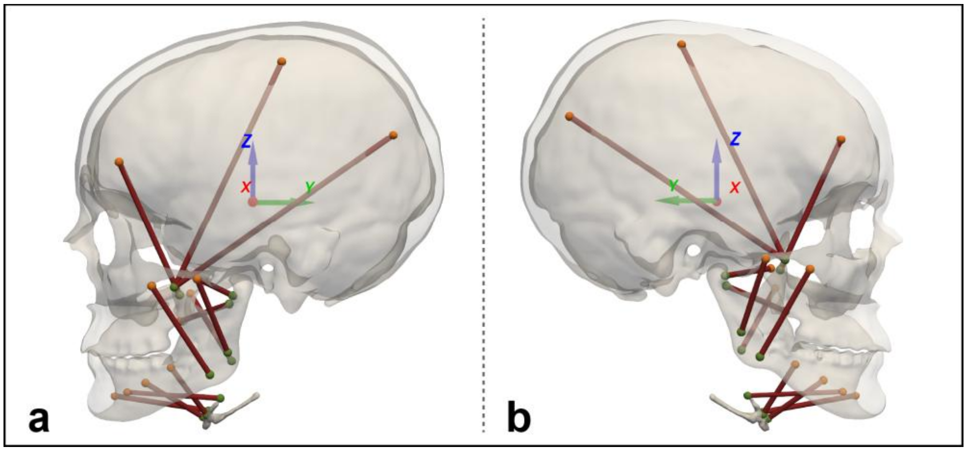

Fig. 3: Visualization of the Class II patient post-op muscle attachment sites **(a)** Left side attachment sites and **(b)** Right side attachment sites. The cranium and mandible are rendered transparently for visualization purposes. Orange markers indicate the attachment site 1, and green markers indicate the attachment site 2 in **Table 7**.

Muscle	Attachment site 1	$X\ (mm)$	$Y\ (mm)$	$Z\ (mm)$	Attachment site 2	$X\ (mm)$	$Y\ (mm)$	$Z\ (mm)$
L Anterior Temporalis	Cranium	52.4	-38.5	18.0	Mandible	39.1	-23.0	-42.6
R Anterior Temporalis	Cranium	-52.2	-38.6	18.2	Mandible	-41.9	-21.3	-40.3
L Middle Temporalis	Cranium	66.0	18.3	55.2	Mandible	43.0	-26.8	-33.6
R Middle Temporalis	Cranium	-65.4	18.4	55.3	Mandible	-45.4	-24.4	-28.6
L Posterior Temporalis	Cranium	59.0	73.8	28.7	Mandible	43.0	-26.8	-33.6
R Posterior Temporalis	Cranium	-58.5	73.9	28.7	Mandible	-45.4	-24.4	-28.6
L Superficial Masseter	Cranium	47.8	-37.1	-38.0	Mandible	39.5	-18.6	-74.3
R Superficial Masseter	Cranium	-47.8	-36.6	-38.6	Mandible	-42.7	-18.6	-74.2
L Deep Masseter	Cranium	57.4	-16.7	-27.4	Mandible	43.0	-5.5	-58.3
R Deep Masseter	Cranium	-58.0	-16.7	-26.2	Mandible	-46.4	-5.3	-58.4
L Medial Pterygoid	Cranium	23.3	-19.8	-35.6	Mandible	36.3	-8.5	-65.7
R Medial Pterygoid	Cranium	-21.2	-19.6	-34.7	Mandible	-39.1	-8.4	-65.6
L Inferior Lateral Pterygoid	Cranium	23.1	-25.3	-30.8	Mandible	46.4	-3.1	-29.6
R Inferior Lateral Pterygoid	Cranium	-21.1	-23.9	-30.4	Mandible	-47.6	-1.3	-28.4
L Superior Lateral Pterygoid	Cranium	27.7	-23.3	-19.6	Mandible	47.8	-1.8	-24.6
R Superior Lateral Pterygoid	Cranium	-27.7	-23.5	-17.8	Mandible	-51.3	-0.8	-23.0
L Anterior Digastric	Mandible	7.5	-58.7	-97.3	Hyoid	13.3	-11.6	-89.3
R Anterior Digastric	Mandible	-10.4	-59.0	-97.1	Hyoid	-11.9	-11.9	-89.8
L Anterior Mylohyoid	Mandible	12.9	-51.5	-88.9	Hyoid	3.9	-21.3	-97.4
R Anterior Mylohyoid	Mandible	-16.5	-50.3	-88.3	Hyoid	-2.1	-21.1	-96.9
L Posterior Mylohyoid	Mandible	17.9	-38.8	-74.4	Hyoid	9.9	-17.9	-95.6
R Posterior Mylohyoid	Mandible	-22.0	-33.9	-73.0	Hyoid	-7.3	-17.7	-95.3
L Geniohyoid	Mandible	1.7	-61.1	-102.2	Hyoid	3.9	-18.2	-93.3
R Geniohyoid	Mandible	-4.5	-61.3	-101.8	Hyoid	-1.9	-17.7	-93.1

Table 8: Muscle attachment sites for the Class III patient pre-op (L, left; R, right)

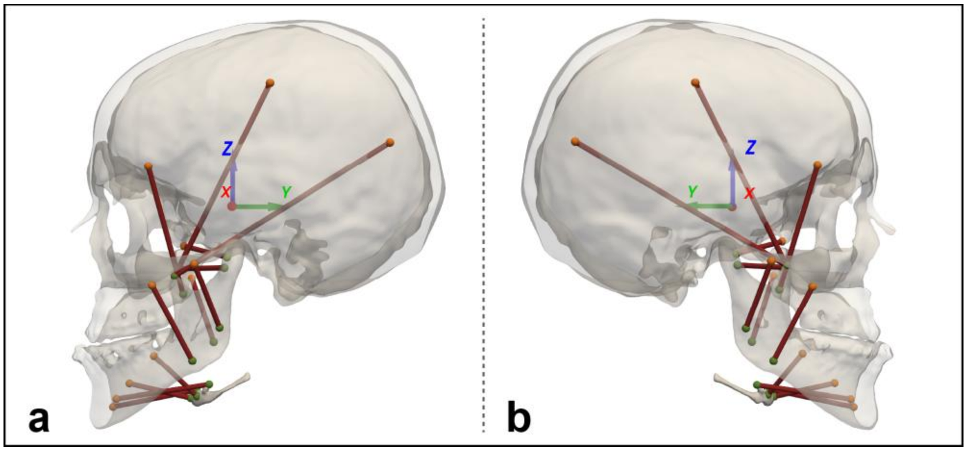

Fig. 4: Visualization of the Class III patient pre-op muscle attachment sites **(a)** Left side attachment sites and **(b)** Right side attachment sites. The cranium and mandible are rendered transparently for visualization purposes. Orange markers indicate the attachment site 1, and green markers indicate the attachment site 2 in **Table 8**.

Muscle	Attachment site 1	$X\ (mm)$	$Y\ (mm)$	$Z\ (mm)$	Attachment site 2	$X\ (mm)$	$Y\ (mm)$	$Z\ (mm)$
L Anterior Temporalis	Cranium	52.4	-38.5	18.0	Mandible	38.9	-18.7	-42.9
R Anterior Temporalis	Cranium	-52.2	-38.6	18.2	Mandible	-43.0	-18.5	-36.8
L Middle Temporalis	Cranium	66.0	18.3	55.2	Mandible	42.7	-24.7	-36.0
R Middle Temporalis	Cranium	-65.4	18.4	55.3	Mandible	-46.4	-23.8	-29.3
L Posterior Temporalis	Cranium	59.0	73.8	28.7	Mandible	42.7	-24.7	-36.0
R Posterior Temporalis	Cranium	-58.5	73.9	28.7	Mandible	-46.4	-23.8	-29.3
L Superficial Masseter	Cranium	47.1	-37.7	-38.3	Mandible	41.1	-10.8	-74.1
R Superficial Masseter	Cranium	-47.1	-37.1	-38.5	Mandible	-43.3	-12.7	-71.8
L Deep Masseter	Cranium	57.0	-18.4	-27.4	Mandible	44.0	-0.2	-59.5
R Deep Masseter	Cranium	-58.0	-17.3	-25.3	Mandible	-44.9	-3.0	-58.7
L Medial Pterygoid	Cranium	19.5	-28.5	-40.8	Mandible	37.7	-1.3	-65.6
R Medial Pterygoid	Cranium	-18.9	-29.5	-41.1	Mandible	-38.3	-6.5	-65.1
L Inferior Lateral Pterygoid	Cranium	23.1	-25.3	-30.8	Mandible	46.2	-1.8	-29.3
R Inferior Lateral Pterygoid	Cranium	-21.1	-23.9	-30.4	Mandible	-48.2	-1.6	-28.7
L Superior Lateral Pterygoid	Cranium	27.7	-23.3	-19.6	Mandible	46.9	-0.5	-24.1
R Superior Lateral Pterygoid	Cranium	-27.7	-23.5	-17.8	Mandible	-49.5	0.1	-23.2
L Anterior Digastric	Mandible	9.4	-53.2	-99.8	Hyoid	13.3	-11.6	-89.3
R Anterior Digastric	Mandible	-8.2	-54.1	-99.2	Hyoid	-11.9	-11.9	-89.8
L Anterior Mylohyoid	Mandible	15.8	-43.6	-87.5	Hyoid	3.9	-21.3	-97.4
R Anterior Mylohyoid	Mandible	-13.4	-46.4	-87.2	Hyoid	-2.1	-21.1	-96.9
L Posterior Mylohyoid	Mandible	20.8	-28.4	-73.6	Hyoid	9.9	-17.9	-95.6
R Posterior Mylohyoid	Mandible	-21.0	-27.0	-72.5	Hyoid	-7.3	-17.7	-95.3
L Geniohyoid	Mandible	4.2	-56.0	-104.1	Hyoid	3.9	-18.2	-93.3
R Geniohyoid	Mandible	-2.6	-56.7	-104.2	Hyoid	-1.9	-17.7	-93.1

Table 9: Muscle attachment sites for the Class III patient post-op (L, left; R, right)

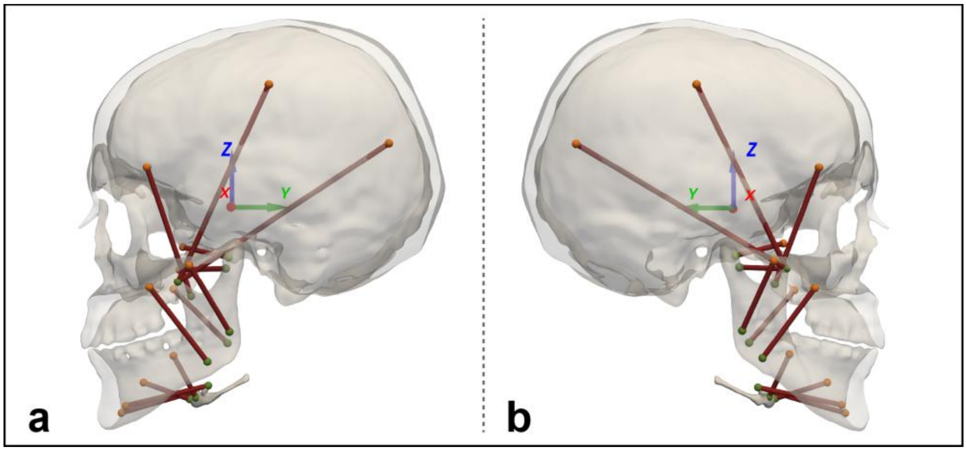

Fig. 5: Visualization of the Class III patient post-op muscle attachment sites **(a)** Left side attachment sites and **(b)** Right side attachment sites. The cranium and mandible are rendered transparently for visualization purposes. Orange markers indicate the attachment site 1, and green markers indicate the attachment site 2 in **Table 9**.

Ligament	Attachment site 1	$X\ (mm)$	$Y\ (mm)$	$Z\ (mm)$	Attachment site 2	$X\ (mm)$	$Y\ (mm)$	$Z\ (mm)$
L Temporomandibular	Cranium	51.2	-22.4	-0.9	Mandible	47.5	-14.8	-10.9
R Temporomandibular	Cranium	-52.8	-22.7	1.6	Mandible	-51.6	-14.9	-11.1
L Sphenomandibular	Cranium	29.4	-19.6	-0.3	Mandible	38.2	-32.7	-31.6
R Sphenomandibular	Cranium	-31.8	-17.5	-1.3	Mandible	-40.2	-31.8	-31.9
L Stylomandibular	Cranium	36.1	-5.5	-13.8	Mandible	38.7	-17.0	-43.3
R Stylomandibular	Cranium	-38.6	-3.6	-11.8	Mandible	-40.8	-16.9	-42.4
L Anterior Disc	L Disc	43.8	-19.8	-1.3	Mandible	43.3	-16.6	-6.9
R Anterior Disc	R Disc	-47.1	-17.7	2.0	Mandible	-47.3	-17.5	-4.0
L Medial Disc	L Disc	38.5	-9.3	-0.7	Mandible	38.6	-7.9	-4.4
R Medial Disc	R Disc	-40.5	-9.8	3.4	Mandible	-41.0	-8.9	0.6
L Lateral Disc	L Disc	51.6	-18.1	-1.4	Mandible	51.4	-18.1	-2.5
R Lateral Disc	R Disc	-54.6	-17.0	0.6	Mandible	-54.5	-17.4	-2.5
L Posterior Inferior Disc	L Disc	45.4	-8.0	0.2	Mandible	45.4	-9.1	-4.6
R Posterior Inferior Disc	R Disc	-48.7	-8.7	3.6	Mandible	-50.1	-10.1	-1.0
L Posterior Superior Disc	L Disc	45.4	-8.0	0.2	Cranium	44.0	-3.7	-4.7
R Posterior Superior Disc	R Disc	-48.7	-8.7	3.6	Cranium	-49.1	-5.7	2.1

Table 10: Ligament attachment sites for the Class I volunteer (L, left; R, right). When the attachment site is located on the discs, the coordinates represent the centroid of a distributed FE attachment, where the applied force is spread over approximately 20 nodes per ligament[1].

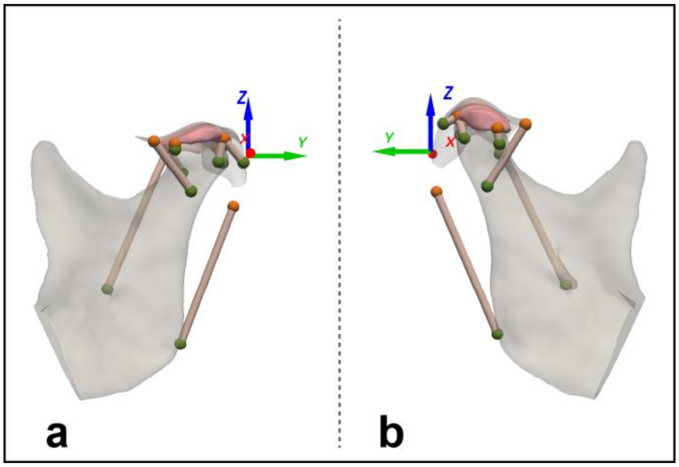

Fig. 6: Visualization of the Class I volunteer ligament attachment sites **(a)** Left side attachment sites and **(b)** Right side attachment sites. The glenoid fossa and part of the mandible are rendered transparent for visualization purposes. Orange markers indicate the attachment site 1, and green markers indicate the attachment site 2 in **Table 10**.

Ligament	Attachment site 1	$X\ (mm)$	$Y\ (mm)$	$Z\ (mm)$	Attachment site 2	$X\ (mm)$	$Y\ (mm)$	$Z\ (mm)$
L Temporomandibular	Cranium	53.9	-16.7	-37.3	Mandible	45.4	-3.0	-46.7
R Temporomandibular	Cranium	-57.8	-14.6	-29.6	Mandible	-46.5	-0.9	-45.2
L Sphenomandibular	Cranium	26.8	-6.8	-38.9	Mandible	37.5	-15.7	-63.8
R Sphenomandibular	Cranium	-29.4	-5.3	-34.3	Mandible	-40.2	-11.3	-61.7
L Stylomandibular	Cranium	38.7	5.9	-42.1	Mandible	37.5	-6.7	-72.6
R Stylomandibular	Cranium	-41.5	9.5	-36.9	Mandible	-40.8	-3.9	-70.6
L Anterior Disc	L Disc	41.2	-10.0	-37.7	Mandible	42.2	-6.0	-39.3
R Anterior Disc	R Disc	-42.2	-8.3	-36.7	Mandible	-42.4	-6.1	-38.0
L Medial Disc	L Disc	33.9	-3.3	-35.5	Mandible	33.6	-2.2	-38.2
R Medial Disc	R Disc	-35.4	-1.3	-33.7	Mandible	-35.8	-1.1	-35.7
L Lateral Disc	L Disc	49.3	-5.2	-35.4	Mandible	49.7	-5.3	-40.6
R Lateral Disc	R Disc	-50.3	-4.4	-32.7	Mandible	-51.0	-3.7	-36.5
L Posterior Inferior Disc	L Disc	42.9	0.9	-33.2	Mandible	42.9	0.5	-40.6
R Posterior Inferior Disc	R Disc	-44.3	2.7	-32.3	Mandible	-43.9	2.9	-38.1
L Posterior Superior Disc	L Disc	42.9	0.9	-33.2	Cranium	43.0	5.5	-37.9
R Posterior Superior Disc	R Disc	-44.3	2.7	-32.3	Cranium	-44.9	9.9	-32.5

Table 11: Ligament attachment sites for the Class II patient pre-op (L, left; R, right). When the attachment site is located on the discs, the coordinates represent the centroid of a distributed FE attachment, where the applied force is spread over approximately 20 nodes per ligament[1].

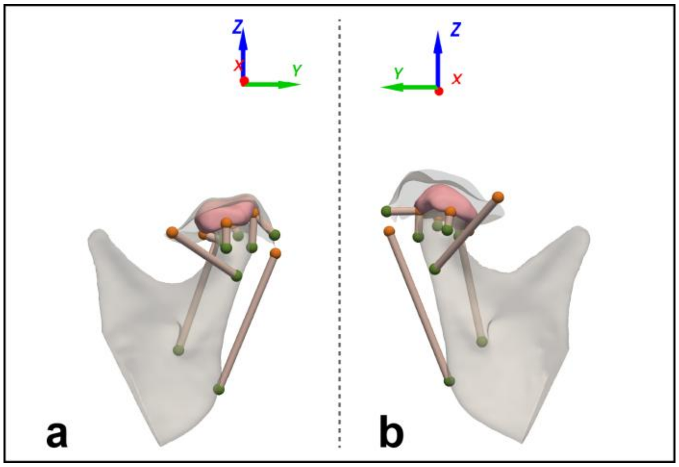

Fig. 7: Visualization of the Class II patient pre-op ligament attachment sites **(a)** Left side attachment sites and **(b)** Right side attachment sites. The glenoid fossa and part of the mandible are rendered transparent for visualization purposes. Orange markers indicate the attachment site 1, and green markers indicate the attachment site 2 in **Table 11**.

Ligament	Attachment site 1	$X\ (mm)$	$Y\ (mm)$	$Z\ (mm)$	Attachment site 2	$X\ (mm)$	$Y\ (mm)$	$Z\ (mm)$
L Temporomandibular	Cranium	53.9	-16.7	-36.9	Mandible	45.8	-2.9	-46.7
R Temporomandibular	Cranium	-55.2	-12.5	-29.8	Mandible	-46.1	0.9	-41.4
L Sphenomandibular	Cranium	27.1	-4.5	-36.5	Mandible	34.8	-17.1	-63.5
R Sphenomandibular	Cranium	-30.6	-5.3	-33.6	Mandible	-40.4	-17.6	-58.2
L Stylomandibular	Cranium	38.8	5.9	-41.6	Mandible	34.2	-6.1	-71.5
R Stylomandibular	Cranium	-41.4	9.6	-36.2	Mandible	-43.2	-7.0	-65.0
L Anterior Disc	L Disc	41.7	-11.0	-38.4	Mandible	43.0	-7.1	-39.6
R Anterior Disc	R Disc	-40.8	-6.5	-34.1	Mandible	-43.0	-2.9	-35.5
L Medial Disc	L Disc	34.9	-3.8	-34.3	Mandible	35.2	-3.1	-36.3
R Medial Disc	R Disc	-35.6	0.0	-31.0	Mandible	-36.1	0.1	-34.1
L Lateral Disc	L Disc	50.3	-6.9	-34.2	Mandible	50.8	-7.0	-42.5
R Lateral Disc	R Disc	-50.1	-2.7	-29.7	Mandible	-50.3	-2.6	-34.6
L Posterior Inferior Disc	L Disc	42.6	-0.9	-31.9	Mandible	42.8	-0.1	-39.8
R Posterior Inferior Disc	R Disc	-44.5	4.4	-29.1	Mandible	-44.2	4.2	-35.3
L Posterior Superior Disc	L Disc	42.6	-0.9	-31.9	Cranium	43.3	5.2	-38.0
R Posterior Superior Disc	R Disc	-44.5	4.4	-29.1	Cranium	-45.4	9.6	-32.4

Table 12: Ligament attachment sites for the Class II patient post-op (L, left; R, right). When the attachment site is located on the discs, the coordinates represent the centroid of a distributed FE attachment, where the applied force is spread over approximately 20 nodes per ligament[1].

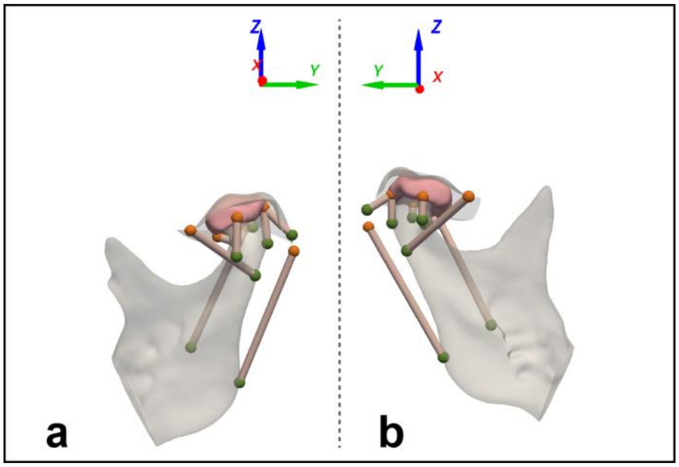

Fig. 8: Visualization of the Class II patient post-op ligament attachment sites **(a)** Left side attachment sites and **(b)** Right side attachment sites. The glenoid fossa and part of the mandible are rendered transparent for visualization purposes. Orange markers indicate the attachment site 1, and green markers indicate the attachment site 2 in **Table 12**.

Ligament	Attachment site 1	$X\ (mm)$	$Y\ (mm)$	$Z\ (mm)$	Attachment site 2	$X\ (mm)$	$Y\ (mm)$	$Z\ (mm)$
L Temporomandibular	Cranium	57.4	-7.4	-26.1	Mandible	51.4	1.0	-36.5
R Temporomandibular	Cranium	-58.0	-6.0	-25.8	Mandible	-52.4	2.9	-33.9
L Sphenomandibular	Cranium	30.5	-2.5	-26.0	Mandible	39.1	-16.2	-60.4
R Sphenomandibular	Cranium	-30.8	1.0	-26.7	Mandible	-43.0	-13.7	-55.7
L Stylomandibular	Cranium	38.6	14.3	-33.8	Mandible	38.3	-0.5	-60.7
R Stylomandibular	Cranium	-40.4	15.8	-34.9	Mandible	-41.9	0.7	-57.2
L Anterior Disc	L Disc	48.4	-2.7	-19.4	Mandible	47.2	-2.4	-27.5
R Anterior Disc	R Disc	-49.9	-1.7	-20.1	Mandible	-49.6	-0.9	-24.9
L Medial Disc	L Disc	38.1	3.5	-19.8	Mandible	38.7	4.1	-19.7
R Medial Disc	R Disc	-40.4	6.0	-18.5	Mandible	-41.0	6.0	-19.8
L Lateral Disc	L Disc	57.0	-1.1	-22.9	Mandible	57.0	-1.4	-26.5
R Lateral Disc	R Disc	-58.4	-0.1	-23.0	Mandible	-58.4	0.5	-24.2
L Posterior Inferior Disc	L Disc	51.7	5.2	-20.3	Mandible	50.5	5.3	-28.8
R Posterior Inferior Disc	R Disc	-52.0	7.3	-19.1	Mandible	-51.7	6.2	-28.5
L Posterior Superior Disc	L Disc	51.7	5.2	-20.3	Cranium	50.4	10.3	-23.2
R Posterior Superior Disc	R Disc	-52.0	7.3	-19.1	Cranium	-51.7	11.1	-22.2

Table 13: Ligament attachment sites for the Class III patient pre-op (L, left; R, right). When the attachment site is located on the discs, the coordinates represent the centroid of a distributed FE attachment, where the applied force is spread over approximately 20 nodes per ligament[1].

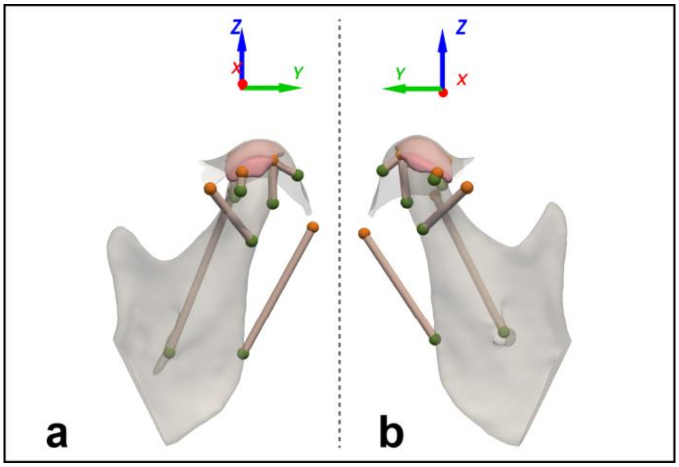

Fig. 9: Visualization of the Class III patient pre-op ligament attachment sites **(a)** Left side attachment sites and **(b)** Right side attachment sites. The glenoid fossa and part of the mandible are rendered transparent for visualization purposes. Orange markers indicate the attachment site 1, and green markers indicate the attachment site 2 in **Table 13**.

Ligament	Attachment site 1	$X\ (mm)$	$Y\ (mm)$	$Z\ (mm)$	Attachment site 2	$X\ (mm)$	$Y\ (mm)$	$Z\ (mm)$
L Temporomandibular	Cranium	57.4	-7.4	-26.1	Mandible	51.3	1.7	-36.6
R Temporomandibular	Cranium	-58.1	-5.8	-24.7	Mandible	-52.8	2.9	-33.5
L Sphenomandibular	Cranium	30.4	-2.5	-25.0	Mandible	39.5	-7.2	-59.2
R Sphenomandibular	Cranium	-30.8	0.8	-26.7	Mandible	-39.8	-9.1	-55.2
L Stylomandibular	Cranium	38.6	14.6	-32.7	Mandible	40.0	5.1	-61.3
R Stylomandibular	Cranium	-40.4	16.0	-34.0	Mandible	-41.7	1.2	-59.2
L Anterior Disc	L Disc	47.5	-2.3	-19.6	Mandible	47.4	-1.5	-27.2
R Anterior Disc	R Disc	-50.6	-2.0	-20.6	Mandible	-49.5	-0.4	-24.9
L Medial Disc	L Disc	38.2	3.8	-18.7	Mandible	38.3	4.5	-19.1
R Medial Disc	R Disc	-40.3	4.4	-18.6	Mandible	-40.4	5.1	-20.2
L Lateral Disc	L Disc	56.9	-0.9	-23.8	Mandible	57.0	-1.4	-26.5
R Lateral Disc	R Disc	-58.3	0.5	-23.2	Mandible	-58.5	0.5	-25.4
L Posterior Inferior Disc	L Disc	50.7	5.3	-19.0	Mandible	50.6	5.6	-28.9
R Posterior Inferior Disc	R Disc	-52.4	7.1	-18.3	Mandible	-51.7	6.3	-28.6
L Posterior Superior Disc	L Disc	50.7	5.3	-19.0	Cranium	50.0	10.6	-23.1
R Posterior Superior Disc	R Disc	-52.4	7.1	-18.3	Cranium	-50.3	11.9	-23.2

Table 14: Ligament attachment sites for the Class III patient post-op (L, left; R, right). When the attachment site is located on the discs, the coordinates represent the centroid of a distributed FE attachment, where the applied force is spread over approximately 20 nodes per ligament[1].

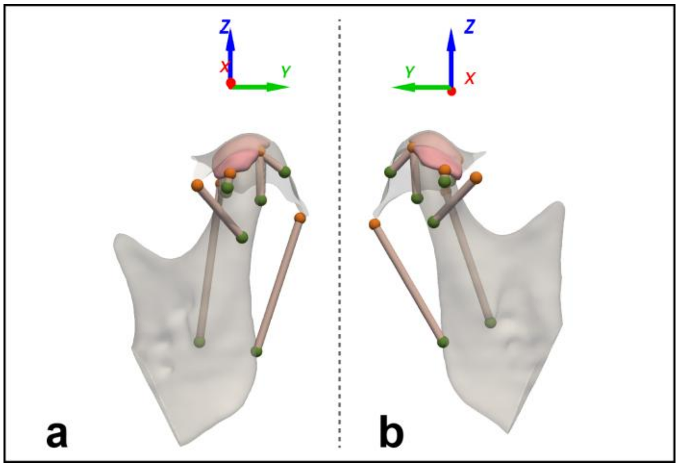

Fig. 10: Visualization of the Class III patient post-op ligament attachment sites **(a)** Left side attachment sites and **(b)** Right side attachment sites. The glenoid fossa and part of the mandible are rendered transparent for visualization purposes. Orange markers indicate the attachment site 1, and green markers indicate the attachment site 2 in **Table 14**.

References

1. Sagl, B., M. Schmid-Schwap, E. Piehslinger, M. Kundi, and I. Stavness. A Dynamic Jaw Model With a Finite-Element Temporomandibular Joint. Front Physiol 10:, 2019. https://doi.org/10.3389/fphys.2019.01156

2. Peck, C. C., G. E. J. Langenbach, and A. G. Hannam. Dynamic simulation of muscle and articular properties during human wide jaw opening. 2000.at <www.elsevier.com/locate/archoralbio>

3. Buchaillard, S., P. Perrier, and Y. Payan. A biomechanical model of cardinal vowel production: Muscle activations and the impact of gravity on tongue positioning. J Acoust Soc Am 126:2033–2051, 2009. https://doi.org/10.1121/1.3204306

Online Resource 3

Schematic of the morphometric measurements presented in the manuscript's Table 3. LIBA, left ipsilateral biting arm; LCBA, left contralateral biting arm; RIBA, right ipsilateral biting arm; RCBA, right contralateral biting arm; LCT, left condyle top; LFP, left first premolar; RCT, right condyle top; RFP, right first premolar; LCA, left condyle area; RCA, right condyle area

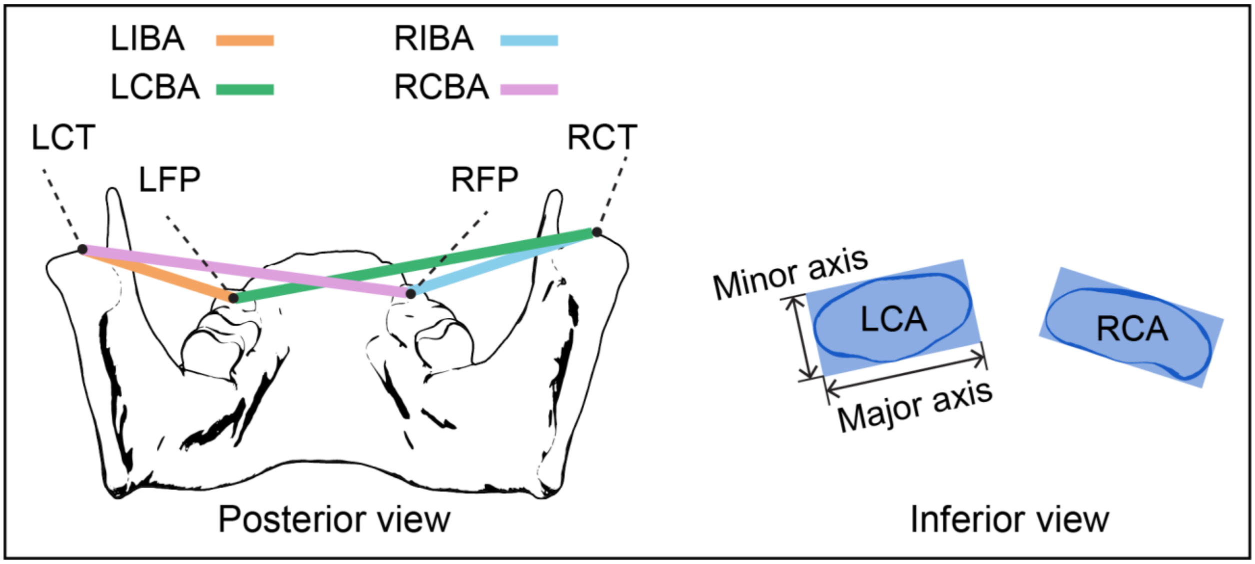

Supplement: Supplementary Information [file NIHMS2157921-supplement-Supplementary_Information.pdf]
